# Rigidly connected multispecific artificial binders with adjustable geometries

**DOI:** 10.1038/s41598-017-11472-x

**Published:** 2017-09-11

**Authors:** Yufan Wu, Alexander Batyuk, Annemarie Honegger, Fabian Brandl, Peer R. E. Mittl, Andreas Plückthun

**Affiliations:** 1 Department of Biochemistry, University of Zürich, Winterthurerstrasse 190, CH-8057 Zürich, Switzerland; 2 Present Address: Paul Scherrer Institute, OFLC/106, 5232 Villigen PSI, Switzerland; 3 Present Address: Linac Coherent Light Source, SLAC National Accelerator Laboratory, 2575, Sand Hill Road, Menlo Park, CA 94025 USA

## Abstract

Multivalent binding proteins can gain biological activities beyond what is inherent in the individual binders, by bringing together different target molecules, restricting their conformational flexibility or changing their subcellular localization. In this study, we demonstrate a method to build up rigid multivalent and multispecific scaffolds by exploiting the modular nature of a repeat protein scaffold and avoiding flexible linkers. We use DARPins (Designed Ankyrin Repeat Proteins), synthetic binding proteins based on the Ankyrin-repeat protein scaffold, as binding units. Their ease of *in vitro* selection, high production yield and stability make them ideal specificity-conferring building blocks for the design of more complex constructs. C- and N-terminal DARPin capping repeats were re-designed to be joined by a shared helix in such a way that rigid connector modules are formed. This allows us to join two or more DARPins in predefined geometries without compromising their binding affinities and specificities. Nine connector modules with distinct geometries were designed; for eight of these we were able to confirm the structure by X-ray crystallography, while only one did not crystallize. The bispecific constructs were all able to bind both target proteins simultaneously.

## Introduction

In the past decade, protein binders based on various scaffolds and selected by display methods from gene libraries randomized in specific positions have been developed as an alternative to monoclonal antibody technology. Different scaffolds offer specific advantages and disadvantages^1^: natural and fully synthetic antibody Fab (~50 kDa) and single-chain (scFv ~25 kDa) libraries mimic the diversity of natural antibody repertoires and yield binding modules similar to those generated by cloning of Fab or scFv fragments from specific monoclonal antibodies. Nanobodies (~12 kDa) mimic camelid V_HH_ domains and shark NAR variable domains, and are either derived from natural repertoires or from synthetic libraries based on humanized frameworks. Adnectins (~10 kDa) are based on the fibronectin type III domain scaffold, anticalins (~20 kDa) on the lipocalin structure, fynomers (~7.5 kDa) are derived from SH3 domains, affibodies (~7 kDa) are a three-helix bundle derived from a domain of protein A, knottins (~3–4 kDa) utilize a cysteine knot (for a recent review of alternative scaffolds in therapeutic use or development, see ref. 2).

Designed ankyrin repeat proteins (DARPins) represent a class of small, highly stable artificial binding proteins based on a repeat protein scaffold^3–5^, which have been developed for biochemical research, diagnostics and clinical use (reviewed in ref. 6). They can be produced with high yields in *E. coli*, as they fold efficiently and do not rely on disulfide bridges for stability. Tightly packed repeats form a continuous hydrophobic core that is shielded from the solvent by specialized N- and C-terminal capping repeats (N-cap and C-cap). In DARPin libraries, six positions of each 33-amino-acid internal repeat are randomized. In selected binders, their surface forms a continuous paratope, stretching over typically two (termed N2C, ~15 kDa) to three (N3C, ~18 kDa) internal repeats. DARPin stability increases with increasing number of repeats: the melting temperature of the non-randomized DARPin scaffold increases from 60 °C for N1C (one internal repeat), 90 °C for N2C (two internal repeats) to >100 °C for N3C (three internal repeats) and larger DARPins. The midpoint of guanidinium hydrochloride denaturation changes from 1.4 M (N1C), 4.1 M (N2C), 5.6 M (N3C), to >8 M (N4C) and even higher for even larger scaffolds^7^. Unfolding of the C-terminal capping repeat at lower temperatures or guanidinium concentrations in the original design was abolished by the introduction of stabilizing mutations in the C-cap^8^. A consequence of this molecular architecture, forming the basis of the present study, is the fact that the DARPin can be rigidly extended on either end. Various selection methods such as ribosome display^5^, phage display^9^ and yeast surface display^10^ have been used to select high-affinity binders from DARPin libraries.

A high-throughput technology has been developed that allows to select binders against 96 targets in parallel, usually yielding a diverse set of binders against several distinct epitopes on each target. From such a set, multivalent binding proteins can be constructed to achieve biological activities beyond what is inherent in the individual binders, especially if their relative orientation can be controlled. These and many other applications demonstrate the urgent need for efficient structural characterization of DARPin complexes as a basis for understanding their mechanism of action and/or further improving the activity: e.g., DARPins have been engineered to work as intracellular kinase sensors^11^, modulated the biological activities of a caspase^12^, served as starting points for engineering constructs active *in vivo* from biologically inactive individual domains (e.g. HER2-binders^13, 14^), or have been combined into retargeting adaptors for gene transfer vectors (Adenovirus knob clamp^15^).

In a recent study, we have shown that other protein domains starting with an α-helix can be rigidly fused to the C-terminal helix of the capping repeat of a DARPin by forming a shared helix that is embedded along its entire length in at least one of the two domains. The relative orientation of the DARPin and its fusion partner depends on the length of the shared helix, giving rise to a whole series of constructs with different molecular geometries. Due to the modular nature of the DARPin structure, these modified capping repeats can be transferred between different binders without disturbing their binding affinity, as long as the capping repeat itself is not involved in binding. To prove this concept, we had previously designed a series of DARPin-β-lactamase fusions^16^, to be used as DARPin-based crystallization chaperones that exploit the β-lactamase to provide essential crystal contacts and explore molecular geometry as a screening dimension in crystallization trials.

In the current study, we present an extension to this approach: since DARPins start and end with α-helices, two or more DARPins can be joined in a similar manner. They can thus bring together two or more different target molecules in a supramolecular complex of predefined geometry, or can extend the footprint of a selected DARPin on its target to block adjacent interaction sites and can thus serve as a conformational sensor. We generated a series of nine connector modules that rigidly join two DARPins in different, well-defined orientations without obstructing the paratopes to test the validity of this design approach. We succeeded in crystallizing most of the two-DARPin and some of the three-DARPin scaffolds in the absence of target proteins to confirm their geometry, and additionally determined structures of some complexes, confirming the concept.

This rigid DARPin-DARPin fusion strategy also gives us another dimension in crystallization screening: Each DARPin can have different fusion and interaction partners, and each fusion partner can in turn have several different fusion geometries, thereby providing an entire new screening dimension in crystallization trials. Furthermore, this fusion strategy is strengthened by the fortuitous discovery of a particular DARPin paratope that forms strong homophilic crystal contacts in several different relative orientations. We show here that this unliganded DARPin (termed *D12*) can be used in conjunction with any DARPin:target complex to facilitate its crystallization, using the rigid connector modules.

Throughout the manuscript, we apply the following nomenclature: DD and DDD represent constructs with two and three DARPin domains, respectively. DD constructs are named *aaa*_Hxx_*bbb*, where *aaa* and *bbb* are the abbreviated names of the N- and C-terminal DARPin domains and Hxx indicates shared helix (with xx ranging from 02 to 15). DDD constructs are named *aaa*_Hxx_*bbb*_Hyy_*ccc* using the same scheme. In case of complexes, the ligand name (e.g., Maltose-Binding Protein (MBP) or Green Fluorescent Protein (GFP)) is inserted right after the name of the cognate DARPin domain, and a colon separates the DARPin domain from its ligand.

## Results

### Design of rigid DARPin-DARPin fusions

**Figure 1:**
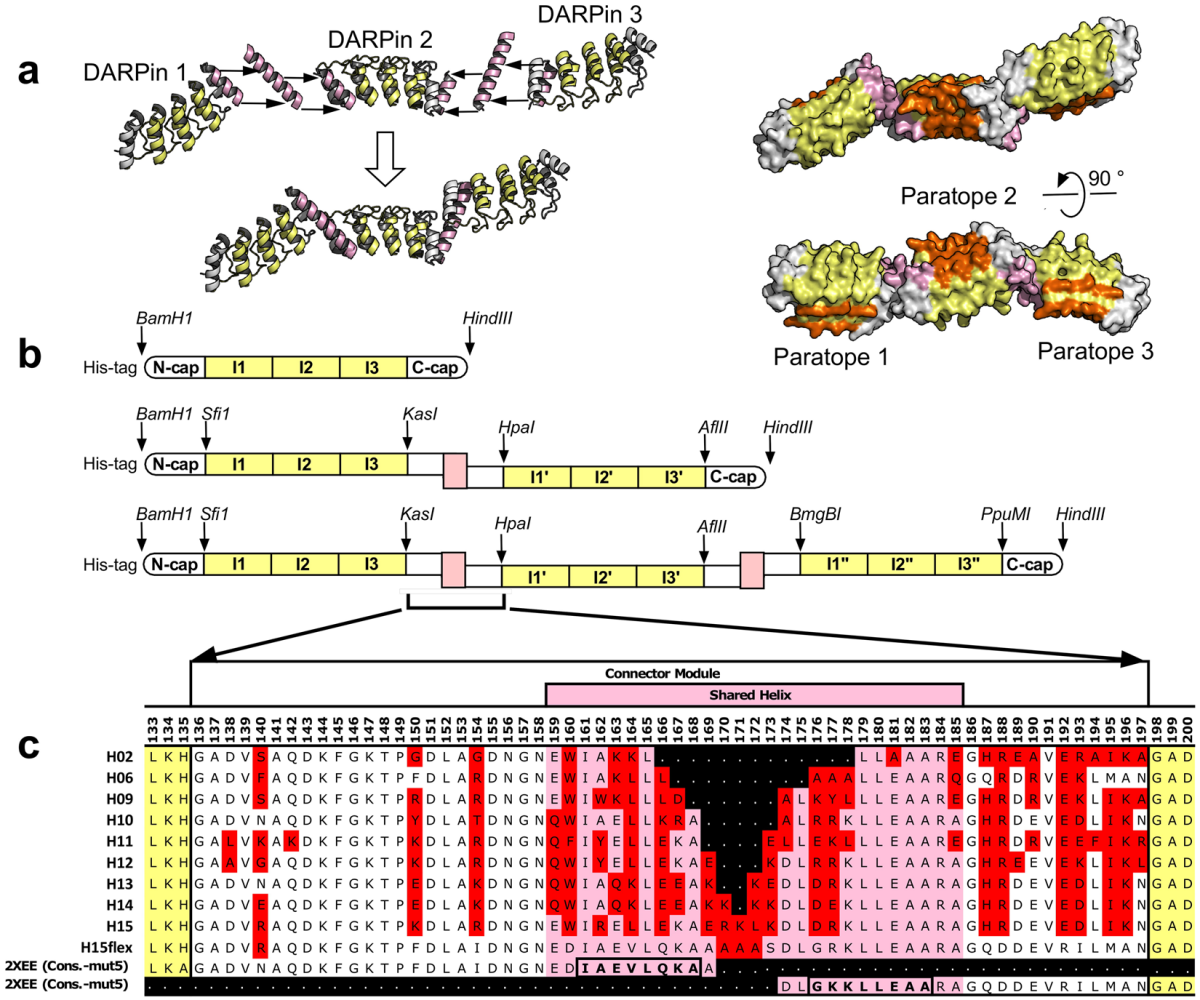
Design of rigid DARPin-DARPin fusions. (**a**) Rigid fusions of two or more DARPins can be generated by joining the C-terminal helix of one DARPin to the N-terminal helix of a second DARPin. The length of the shared helix should optimally be less than the sum of the lengths of the individual helices, ensuring that the helix is embedded in at least one of the two domains along its entire length. The overall geometry of the construct depends on the length of the shared helix. Since the C-and the N-terminal helix of a DARPin run roughly antiparallel to each other, this results in the two paratopes facing in opposite directions, minimizing the probability of target proteins bound to the two paratopes clashing with each other. Capping repeats are shown in white, the terminal helices giving rise to the shared helix in pink, the internal repeats carrying the DARPin paratope in yellow. Residues randomized in the DARPin library, giving rise to the paratope in selected binders, are highlighted in orange. (**b**) Genetic organization of the constructs. The binding specificity of each DARPin is encoded in the sequence of its internal repeats (I1 to I3, etc.). The number of internal repeats can vary, three being most common. A non-randomized spacer repeat can be inserted between the randomized repeats and the connector module to adjust the spacing between the two paratopes and to avoid loss of affinity if the paratope of a selected binder extends onto the capping repeat. To facilitate the exchange of DARPin specificities, unique restriction sites were introduced between internal repeats and connector modules. (**c**) Sequences of the connector modules joining two DARPins. Residues retained from the original capping repeats (taken from a consensus DARPin with stabilized C-cap, mut5, PDB ID: 2XEE^22^) are shown in white and pink, residues changed by Rosetta *fixbb* ^17^ sequence optimization are highlighted in red.

The relative spatial orientation of the paratopes recognizing the ligands of a multivalent or bispecific DD construct is key for its functionality. In our constructs, the rigid helix connector between the DARPin domains determines the angle and distance between the DARPins, controlling the relative spatial orientation of the paratopes. Rigid shared helix connectors were designed using a multi-step procedure: (i) An idealized poly-alanine helix was superimposed on the C-terminal helix of the first DARPin (residues 161–168 for an N3C DARPin containing three internal repeats). (ii) The N-terminal helix of a second DARPin (residues 15–22) was superimposed onto a sliding 8-amino acid segment of that idealized poly-alanine helix (Fig. 1a). The raw models were screened for backbone-backbone clashes (Supplementary Fig. 1), and clashing models were eliminated. (iii) The sequence of the shared helix was designed such that residues interacting with the DARPin cores were maintained. Rosetta *fixbb* ^17^ was then used to optimize the sequence of the connecting module, restricting sequence changes to positions within the original capping repeats (residues 136–197) and the shared helix and to substitutions that made a significant contribution to the total energy of the construct (Fig. 1c). (iv) To facilitate the exchange of DARPin specificities, unique restriction sites were placed at the boundaries between the specificity-determining internal repeats of the DARPins and the rigid shared helix connector modules (Fig. 1b).

**Figure 2:**
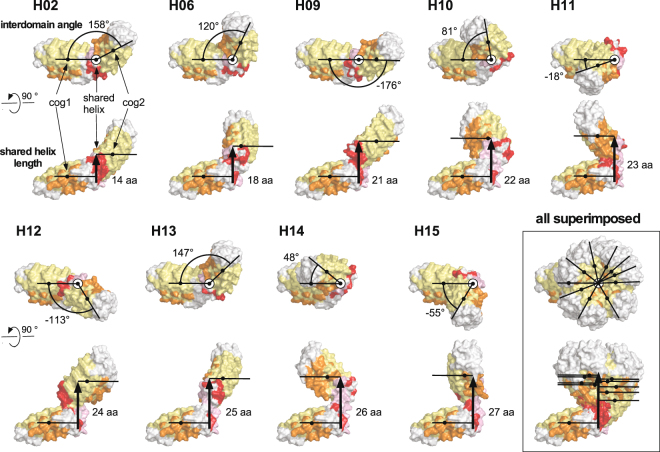
Geometry of the nine shared helix constructs. The relative orientation of the two DARPins in the DD constructs is defined as the pseudo-torsion angle between the centers of gravity (cog) of the internal repeats of the two DARPins around the axis of the connecting helix. The nine DD constructs (H01, H06, H09, H10, H11, H12, H13, H14 and H15) are shown with the N-terminus of the first DARPin to the left. The top view of each construct illustrates the relative domain orientation; the axis of the shared helix is perpendicular to the image plane. The bottom view is rotated by 90°, illustrating the length of the shared helix (shown as black arrow). Capping repeats and connector modules are colored white, internal repeats pale yellow. Randomized residues determining the specificity of each DARPin are highlighted in orange, the shared helix in pink and mutations introduced to stabilize the connector module in red. The last panel shows an overlay of all nine models, illustrating the range of conformation covered by the nine constructs.

This process yielded nine non-clashing constructs with shared helix lengths between 14 and 27 amino acids (DD constructs H02, H06, H09, H10, H11, H12, H13, H14, H15). Different lengths of the shared helix result in different relative domain orientations. These were quantified by a pseudo-torsion angle, which defines the helix as the axis and the two centers of gravity of the DARPin domains (themselves calculated from the Cα atoms of the three internal repeats) (Fig. 2). While constructs with longer shared helices are possible, we restricted the upper helix length to 27 residues, since the probability of local helix unfolding and/or bending increases with helix length. DDD models, containing three DARPins, were generated by superimposing the Cα atoms of the internal repeats of the second DARPin in one DD construct onto the internal repeats of the first DARPin in a second DD construct, generating a total of 81 possible DDD geometries.

Superposition of the DARPin *off7*:MBP complex (Protein Data Bank (PDB) ID: 1SVX^5^) onto each of the DARPins in all constructs showed that in all DD constructs, all paratopes should be able to bind MBP-sized ligands simultaneously without clashing. The same is true for 76 out of 81 DDD constructs. In five constructs (with the helix combinations H11/H10, H11/H14, H12/H06, H15/H10 and H15/H14), MBP binding to the first and third paratope would clash with each other (data not shown). The size of proteins which can be bound simultaneously to the different paratopes within a multivalent construct depends not so much on the distance between the paratopes than on their relative spatial orientation (for illustration, see Supplementary Fig. 2 for models of the nine symmetric DDD constructs with MBP bound to all three DARPin units).

### Expression and Characterization

All DD and DDD constructs used for crystallization trials, carrying DARPins with different specificities, were expressed as soluble proteins in the cytoplasm of *E. coli* XL1-Blue and purified by immobilized metal ion affinity chromatography (IMAC). Each construct yielded 20 to 30 mg of pure protein per liter from non-optimized shake flask cultures. All DD constructs tested were found to retain binding to the respective targets, as analyzed by ELISA (Supplementary Fig. 3a), with signals similar to those of the monovalent DARPins, included as controls. Size exclusion chromatography (SEC) in the absence and presence of target molecules was used to confirm the stability of the ternary complexes and to determine the oligomeric states of the DD fusion constructs and their complexes with the cognate targets, e.g. MBP (42 kDa) and GFP (35 kDa). All DD constructs were monomeric at a concentration of 10 µM on analytical SEC (data not shown). The DD constructs bound their respective targets, as indicated by a shift of the preparative SEC peak from the elution volume of the unliganded DD constructs to that expected for the molecular weight of the complex (Supplementary Fig. 3b). All 9 *off7*_Hxx_*3G124* constructs were able to bind MBP and GFP simultaneously, demonstrating the absence of steric hindrance between the two targets, consistent with the structural models.

### Crystal structures of unliganded DD and DDD constructs

To validate the design, we first determined the crystal structures of the constructs. In this study, we present the structures of a total of fourteen DARPin-DARPin (DD) and DARPin-DARPin-DARPin (DDD) constructs, two of them as complexes with their target proteins. The structures represent eight out of nine different shared helix designs. To facilitate crystallization of the unliganded constructs, we used the internal repeats of DARPin *D12*. This DARPin was originally selected to recognize the V3 loop of human immunodeficiency virus envelope glycoprotein gp120^18^. In the context of DARPin-β-lactamase fusions^16^, we observed that the paratope of this DARPin has a high propensity to form major crystal contacts, leading to crystal formation under a wide variety of conditions. Indeed, constructs *D12*_Hxx_*D12* with H02, H09, H11, H13 and H15 yielded diffraction-quality crystals in space groups *P*2_1_2_1_2_1_, *P*6_1_22, *P*2_1_2_1_2_1_, *C*2 and *P*1, respectively, at resolutions ranging from 1.8 Å to 3.5 Å (Table 1).

**Table 1:** Overview over composition, crystal form, resolution, relative domain orientation, and agreement between design and experimental structures.

PDB ID	Construct	DARPins	Shared helix length	Ligands	Space group	mol/au	Resolution	Angle design	Angle structure	rmsd to model
**5LW2**	DARPin *D12*	*D12*	—	unliganded	*C*2	2	1.8 Å			
**5LE3**	*D12*_H02_*D12*	*D12*/*D12*	14 aa	unliganded	*P*2_1_2_1_2	4	3.5 Å	158	160	1.4
**5LE6**	*D12*_H09_*D12*	*D12*/*D12*	21 aa	unliganded	*P*6_1_22	6	1.8 Å	−176	172	7.0
**5LE4**	*D12*_H11_*D12*	*D12*/*D12*	23 aa	unliganded	*P*2_1_2_1_2_1_	1	2.4 Å	−18	−25	6.4
**5LE7**	*D12*_H13_*D12*	*D12*/*D12*	25 aa	unliganded	*C*2	4	2.1 Å	147	144	1.0
**5LE8**	*D12*_H15_*D12*	*D12*/*D12*	27 aa	unliganded	*P1*	2	1.8 Å	55	64	2.0
**5LEB**	*D12*_H06_*D12*_H06_*D12*	*D12*/*D12*/*D12*	18 aa	unliganded	*C*2	1	2.3 Å	120	134/140	4.1
**5LEC**	*D12*_H12_*D12*_H12_*D12*	*D12*/*D12*/*D12*	24 aa	unliganded	*P*2_1_2_1_2_1_	1	2.5 Å	−113	−94/−113	4.2
**5LED**	*D12*_H12_*D12*_H12_*D12*	*D12*/*D12*/*D12*	24 aa	unliganded	*P*2_1_	1	2.6 Å	−113	−110/−112	1.5
**5LEE**	*D12*_H12_*D12*_H12_*D12*	*D12*/*D12*/*D12*	24 aa	unliganded	*P*3_1_21	1	2.4 Å	−113	−101/−103	2.8
**5LE2**	*D12*_H15_*D12*_H15_*D12*	*D12*/*D12*/*D12*	27 aa	unliganded	*P*2_1_	2	2.4 Å	55	70/66	4.9
**5LE9**	*off7*_H09_*3G124*	*off7*/*3G124*	21 aa	lost (pH 4.6)	*P*4_1_2_1_2	1	1.9 Å	−176	128	6.0
**5LEL**	*off7*:MBP_H10_*3G124*:GFP	*off7*/*3G124*	22 aa	MBP, GFP	*C*2	3*3	3.1 Å	81	113	4.1
**5LEM**	*off7*:MBP_H11_*3G124*:GFP	*off7*/*3G124*	23 aa	MBP, GFP	*P*2_1_2_1_2	1*3	3.0 Å	−18	−18	1.7
**5LEA**	*off7*_H12_*3G124*	*off7*/*3G124*	24 aa	lost (pH 5.0)	*P*2_1_	1	2.4 Å	−113	−114	1.0

**Figure 3:**
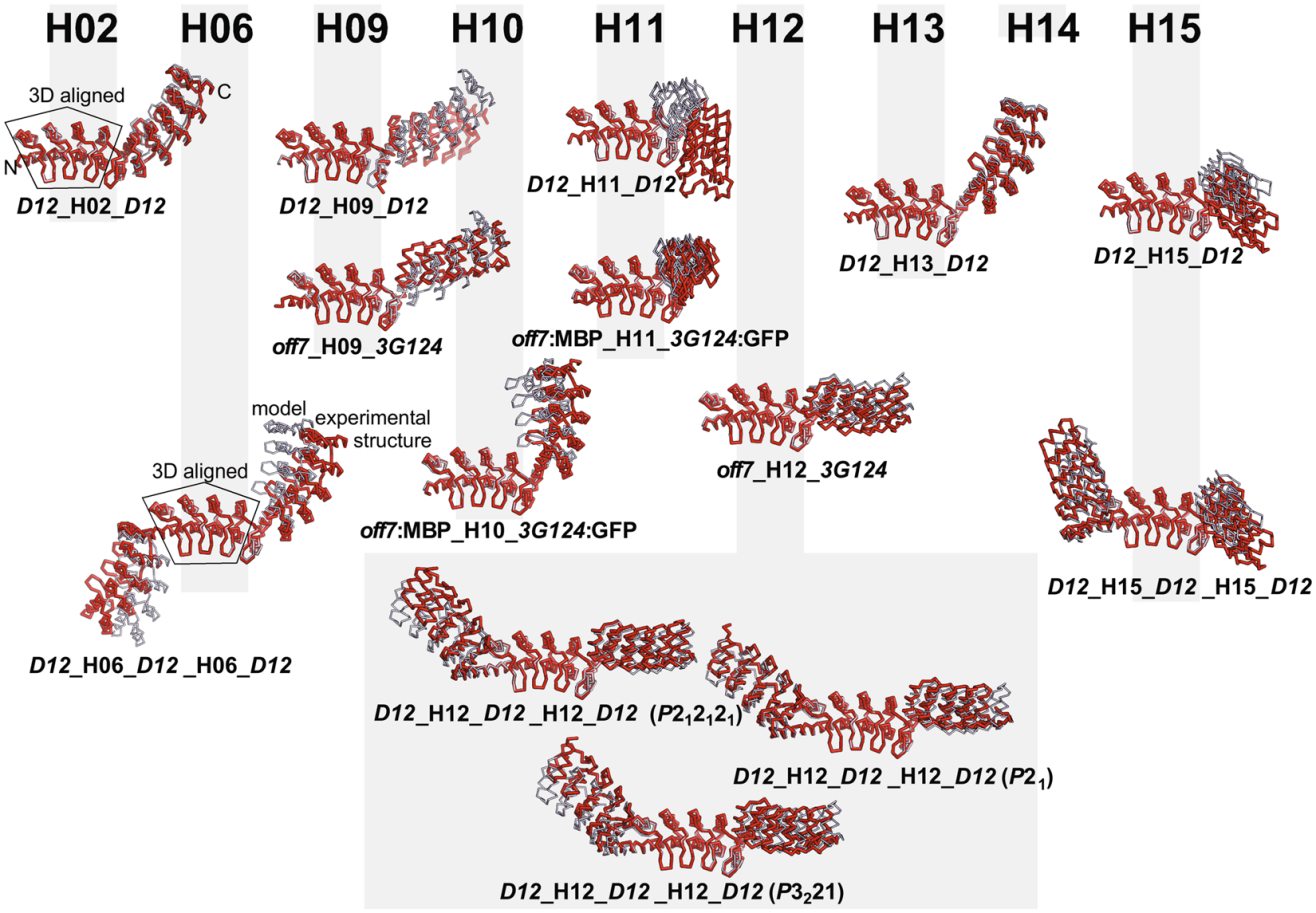
Superposition of design and experimental structures. Models and experimental structures were superimposed by a least-squares fit of the Cα atoms of the three internal repeats of the first DARPin in the case of the two-DARPin constructs, or of the middle DARPin in the case of the three-DARPin constructs, with rmsds of 0.4 ± 0.03 Å. Models are shown in pale blue, experimental structures in red. Rmsd values for the superposition of the whole molecules are listed in Table 1, more details are given in Supplementary Tables 2 to 4. DD constructs are named *aaa*_Hxx_*bbb*, where *aaa* and *bbb* are the abbreviated names of the N- and C-terminal DARPin domains, and Hxx indicates shared helix (with xx ranging from 02 to 15). DDD constructs are named *aaa*_Hxx_*bbb*_Hyy_*ccc* using the same scheme. In case of complex structures, the ligand name (e.g. MBP or GFP) is inserted right after the name of the cognate DARPin domain. The three independent crystal structures of construct *D12*_H12_*D12*_H12_*D12* are distinguished by the space group of the crystals, *P*2_1_, *P*2_1_2_1_2_1_ and *P*3_2_21.

Naming conventions are explained in Fig. 3. In case of complexes, the ligand name (e.g. MBP or GFP) is inserted right after the name of the cognate DARPin domain. A colon separates the DARPin domain from its ligand. The unfused DARPin *D12* has been added as reference. “Angle” refers to the pseudo-torsion angle between the two DARPin domains, as defined in Fig. 2.

For additional details, see Supplemental Tables ST1 (Crystallization conditions, data collection and refinement statistics) and ST2 to ST4 (comparison of models and structures).

The combination of nine different geometries for individual connector modules results in 81 different geometries for triple DARPin fusions. We only tested the nine “symmetric” *D12*_Hxx_*D12*_Hxx_*D12* constructs, with the same connector geometries on either side of the central DARPin domain. *D12*_H06_*D12*_H06_*D12* crystallized in space group *C*2 and was refined at 2.3 Å resolution. *D12*_H12_*D12*_H12_*D12* crystallized under several different conditions; three structures were determined in space groups *P*2_1_2_1_2_1_, *P*2_1_, and *P*3_2_21 at resolutions between 2.4 Å and 2.6 Å. *D12*_H15_*D12*_H15_*D12*, the construct with the longest shared helices, crystallized in space group *P*2_1_ and the structure was refined at 2.4 Å resolution. Symmetric DDD constructs with *D12* domains and H02, H09, H10, H11, H13, and H14 connectors did not yield diffraction-quality crystals upon initial screening.

### Comparing designs and experimental structures

Table 1 summarizes the crystal parameters, relative domain orientations and agreement between models and experimental structures, and Fig. 3 illustrates the agreement between the designed models and experimental structures. Crystallization conditions, refinement statistics, and additional details are listed in Supplementary Tables 1 to 4. Five out of nine DD structures showed good agreement between design and experimental crystal structure with root mean squares deviation (rmsds) of the Cα atoms below 2 Å (Table 1, Fig. 3). This confirms the design strategy and validates the concept of a shared helix as a rigid connector. Thus, we can use this information to predict the relative orientation of binding sites with sufficient accuracy for future functional studies of DD and DDD constructs. In only three out of fourteen structures, dominant crystal contacts, sometimes only under specific crystallization conditions (low pH) significantly perturbed the geometry of the connector module. These deviations are discussed in the Supplementary Experimental Procedures and in Supplementary Fig. 4, and we argue that the structure in solution at neutral pH is likely to be very close to the design.

The structure of *D12*_H12_*D12*_H12_*D12* was determined in three different space groups (*P*2_1_2_1_2_1_, *P*2_1_, and *P*3_2_21), which gives an estimate of the impact of crystal lattice forces on the shape of the molecule. Rmsds between chemically identical *D12*_H12_*D12*_H12_*D12* constructs vary between 1.6 and 2.9 Å. The rmsd for the comparison between design and crystal structure of DDD constructs with H06, H12 and H15 ranges from 1.5 to 4.2 Å. Considering the elongated shape of the DDD molecules, which amplifies the effect of small deviations in the connector, they agree remarkably well with the designs. Differences can be attributed to crystal lattice forces in the different lattices, which are a consequence of different crystallization conditions.

### Crystal structures of ligand-bound DD constructs

In a second series of DD constructs, the N-terminal DARPin *D12* was replaced by *off7*, a high-affinity binder for MBP^5^, while the C-terminal *D12* was replaced by *3G124*, recognizing GFP^19^. For crystallization trials, the ternary complexes of all nine DD fusions with MBP and GFP were purified by preparative size exclusion chromatography. Two DD constructs yielded crystals of the ternary complex: *off7*:MBP_H10_*3G124*:GFP crystallized in space group *C*2 (3.1 Å resolution, 3 complexes/ asymmetric unit (a.u.)) and *off7*:MBP_H11_*3G124*:GFP in space group *P*2_1_2_1_2 (3.0 Å resolution). All three components of the complexes were well-resolved in the electron density maps. Constructs *off7*_H09_*3G124* (crystallized at pH 4.6) and *off7*_H12_*3G124* (crystallized at pH 5.0) diffracted to 1.9 Å and 2.4 Å resolution, respectively. In these structures the ligands were lost from the complex at acidic crystallization conditions (Supplementary Table 1). The other five *off7*_Hxx_*3G124* constructs (with xx equal 02, 06, 13, 14, 15) did not yield diffraction-quality crystals. Crystallization of triple DARPin fusions with MBP and GFP ligands was not attempted.

**Figure 4:**
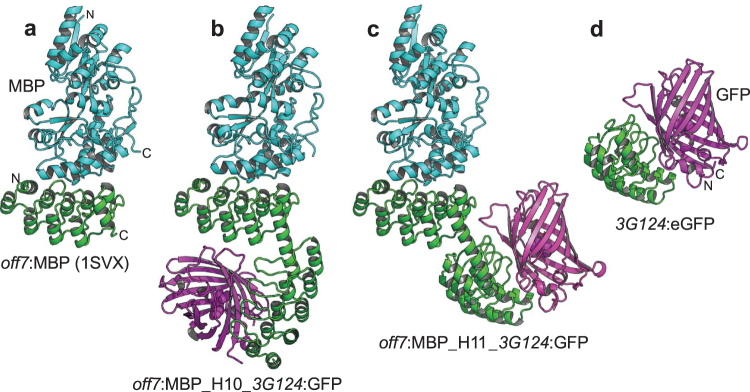
Ternary complexes of DD constructs with two different target proteins. (**a**) Structure of DARPin *off7* in complex with maltose binding protein (MBP) (PDB entry 1SVX,^5^). (**b**) Ternary complex *off*7:MBP_H10_*3G124*:GFP. (**c**) Ternary complex *off7*:MBP_H11_*3G124*:GFP. (**d**) Structure of DARPin *3G124*:eGFP (S. Hansen, unpublished results). Structures a, b and c were superimposed by a least-squares fit of the *off7* internal repeats. Structure d was superimposed on structure c by a least-squares fit of the *3G124* internal repeats.

In the complexes, the interactions between DARPins and target proteins were found to be very similar to the interactions seen in the absence of fusion partners (Fig. 4). This is an important observation, as this reassures that any conclusions derived from complexes of DD and DDD fusions will also be valid for individual DARPins. Therefore, the fusion strategy with the well crystallizing DARPin *D12* can be used to generate structural information of the unfused complex.

### *D12*-mediated crystal contacts

The β-lactamase fusions with DARPin *D12* ^16^ had shown that, while the paratope-paratope interactions of *D12* are weak enough for constructs containing this DARPin to remain monomeric during production and purification, they are strong enough to frequently form extended crystal contacts that have enabled crystallization under a wide variety of conditions. We therefore utilized *D12* to facilitate crystallization in this study and analyzed the crystal contacts formed by *D12* and its fusion constructs. We wanted to elucidate the general features of a molecule such as *D12* that has these favorable crystallization properties.

**Figure 5:**
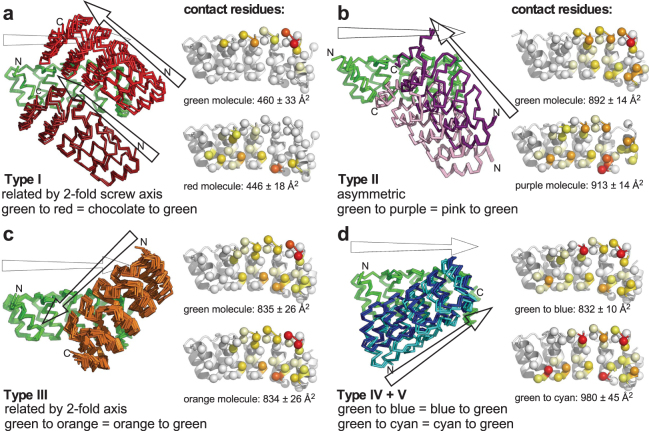
Dominant crystal contacts mediated by the *D12* paratope. Symmetry-related molecules surrounding the asymmetric units of the different DD constructs were generated using the program PyMOL (www.pymol.org) and crystal contacts were analyzed. Panels a–d show the five different classes of interfaces observed. *Left*, relative orientation of interacting DARPin(s) with respect to the green reference DARPin; arrows indicate the direction of the DARPins, from N-term to C-term. In panel d, cyan shows Type IV and dark blue shows type V. *Right*, per-residue surface accessibility of the isolated DARPin units and the pair forming a crystal contact were calculated using the program NACCESS (www.bioinf.manchester.ac.uk/naccess/), and from the difference the contribution of each residue to the interface was calculated and color-coded onto the Cβ atom represented as sphere (red: >100 Å^2^, orange-red: >80 Å^2^, orange: >60 Å^2^, yellow-orange. >40Å^2^, yellow: >20Å^2^, cream: >0 Å^2^, white: not involved in interface). The analysis is described in more detail Supplementary Experimental Procedures, and details of the interactions are presented in Supplementary Fig. 5a–e, a superposition of the five different relative orientations in Supplementary Fig. 5f.

Indeed, in the majority of the *D12*-containing structures analyzed in this study, the *D12* paratope-paratope interaction provided the largest crystal contact, with a total of around 1700 Å^2^ of solvent-accessible surface buried in the interface. Although the paratope-paratope interactions predominantly involve the second and third internal repeats and the adjacent C-cap of the DARPin units involved, the geometry of the interaction in the different structures falls in several distinct classes. For the sake of clarity, in Fig. 5 we show only the two DARPin moieties involved in the contact, aligned by one of the DARPins. The contribution of the individual interface residues to the total surface buried in the interface is shown as a color code on the opened-up interface. Supplementary Fig. 5 offers a more detailed view of the residue interactions involved in interface formation.

#### Interface type I

In the crystal of unliganded DARPin *D12* (details: Table 1), the paratope-paratope interaction is split into two interfaces (i.e., interacting with different molecules) of 498/473 A^2^ and 405/438 Å^2^ of surface buried, equivalent to 71% of all crystal contacts made by *D12*. The brown, green and red molecules shown in Fig. 5a and Supplementary Fig. 5a are related by a two-fold crystallographic screw axis. Additional interfaces are provided by capping repeats and by bridging Ca^2+^ ions. In the *D12*_H06_*D12*_H06_*D12* crystal, both the N-terminal and the C-terminal DARPin units form similar paratope-paratope interfaces with two symmetry-related molecules each. To accommodate the capping repeats packing against the central DARPin, the inter-domain angle in this DDD construct widens from 120° in the model to 140° in the experimental structure.

#### Interface type II

The contacts shown in Fig. 5b are all within the asymmetric unit of *D12*_H09_*D12*. The observed pseudo-torsion angle of 172° is close to the predicted angle of −176°, but the connecting module is partially disordered, such that the C-terminal DARPin is rotated 90° around its long axis. In this orientation, all four *D12* units engage in paratope-paratope interactions, where two DARPin domains form an antiparallel dimer. Two such dimers pack into a barrel-like unit (Supplementary Figs 4a and 5b). The purple molecule in Fig. 5b shows the C-terminal DARPin packing against the green N-terminal DARPin, the pink molecule the N-terminal DARPin packing against the green C-terminal DARPin.

#### Interface type III

This is the most frequently observed paratope-paratope arrangement (Fig. 5c and Supplementary Fig. 5c). While the interface itself shows local two-fold symmetry, this interaction is also seen between non-symmetry-equivalent units, e.g. between the N-terminal and the C-terminal DARPin moiety of two symmetry-related molecules. In *D12*_H12_*D12*_H12_*D12* (*P*3_2_21), each of the three *D12* paratopes is involved in an interaction of this type: The N-terminal DARPin interacts with the C-terminal DARPin of one symmetry-related molecule, the middle DARPin with the middle DARPin of a second one, and the C-terminal one with the N-terminal DARPin of a third symmetry mate. Similarly, in *D12*_H15_*D12*_H15_*D12*, the N-terminal DARPin interacts with the C-terminal DARPin of one symmetry-related molecule, the middle DARPin with the C-terminal DARPin of a second one, and the C-terminal DARPin with the middle DARPin of a third symmetry-related molecule. In *D12*_H12_*D12*_H12_*D12* (*P*2_1_2_1_2_1_) only two of the three *D12* domains are involved in paratope-paratope contacts, while the paratope of the C-terminal *D12* domain contacts the backside of the middle *D12* and the connector module and shared helix linking it to the C-terminal DARPin. *D12*_H15_*D12* makes paratope-paratope contacts of this type both within the asymmetric unit and with symmetry-related molecules.

#### Interface type IV and type V

Two more types of paratope-paratope interactions with local two-fold symmetry are found that are similar to each other but differ from each other by the angle of interaction (Fig. 5d, Supplementary Fig. 5d,e). Type IV is found in *D12*_H02_*D12* within the a.u., while contacts between molecules related by crystallographic symmetry are of type III. Type V (blue) contacts were not found in the molecules described here, but were found in fusions of *D12* with beta-lactamase (DB_04_v3_*D12*; PDB ID: 5AQ7)^16^.

Only three of the *D12* constructs did not rely on paratope-paratope interactions, but instead relied on paratope-capping repeat or paratope-connector module interactions as dominant crystal contacts: *D12*_H11_*D12*, *D12*_H13_*D12* and *D12*_H12_*D12*_H12_*D12* (*P*2_1_). Constructs containing the H14 connector module failed to crystallize. Modeling showed that the most favored paratope-paratope contact of one DARPin would lead to clashes of the second DARPin for *D12*_H14_*D12*, offering a potential explanation for this observation.

In summary, DARPin *D12* can form five predominant types of interaction with its paratope in arrangements with and without local symmetry, compatible with numerous crystal forms, involving both non-polar and charge interactions (Supplementary Fig. 5), while it shows no dimer formation in solution in any fusion protein under the conditions tested.

## Discussion

In this paper, we present a strategy to fuse specific, high-affinity protein binders to obtain multivalent or multispecific scaffolds of predetermined geometry. DARPins are used as building blocks to confer the desired specificity to the construct. A series of rigid connector modules replacing the DARPin capping repeats allow to combine the individual DARPin domains in such a way that their distance and angle can be altered to test different geometries, bringing the binding partners of the DARPins into a defined spatial arrangement. To test the accuracy of the design we determined the experimental structures of different bi- and trivalent constructs.

The key feature of the novel constructs presented in this study is the rigid yet adjustable connection between the DARPin moieties, which becomes possible through the shared helix design — a feature not achievable for most scaffolds. While the simplest way to recruit target proteins into multi-component complexes would be to fuse the target-binding domains by flexible linkers, this obvious strategy will fail to control the spatial orientation of targets, which in many cases is key for the molecular function of the complex. We chose to use α-helices as rigid linkers to connect the DARPin domains, because DARPins possess helices on their N- and C-terminal ends, allowing a tight integration of the connector into the fold of the target-binding domain. The two helices are not just joined end-to-end, but instead the two domains are made to overlap in such a way that the joining helix is stabilized by contacts with the domain cores along its entire length. As a result, the sequence of the joining helix and the contacting domains had to be adapted using the Rosetta software suite^17^.

In total, we designed and tested nine different connector geometries and confirmed the success of the designs by biochemical and biophysical methods. All DD and DDD constructs are well-behaved proteins that can be expressed at high yield from bacterial expression cultures, are monomeric even for binding domains with relatively hydrophobic paratopes, and retain the target-binding affinities of the parental DARPins. Fourteen crystal structures of DD and DDD constructs, covering eight out of nine connector geometries, validate the accuracy of the design. Indeed, for most constructs, experimental crystal structures agree remarkably well with the designs (Table 1 and Fig. 3). A comparison of three structures of the same construct (*D12*_H12_*D12*_H12_*D12*), determined in different crystal lattices, shows that differences between the structures (rmsds up to 3 Å) are caused by crystal lattice forces. The deviations between different crystal forms are of the same magnitude as the deviations between structures and model. The structures of these DDD fusions are very extended in one direction (>110 Å from N- to C-terminus), and thus amplify the effects of small deviations in the inter-domain angles, making the agreement with the designs even more remarkable. For most DD and DDD constructs, the rmsds between design and experimentally determined structures are even lower, confirming the suitability of the design strategy. This validation is particularly important for the design of even larger DARPin-derived scaffold proteins and the interpretation of the different biological effects of DARPins joined in different relative orientations. Within the constructs, the DARPins moieties always keep their intrinsic structure due to their high stability, and in the majority of cases the shared helix stays intact. Only a few structures show a kinked, disordered or dissociated shared helix (e.g. *D12*_H09_*D12*), but even then the DARPins stay fully intact, demonstrating that local perturbations of the structure in the helix region can be accommodated without unfolding the DARPin moieties.

Crystallization of the constructs was facilitated by the discovery of a particular DARPin paratope that appears to frequently make versatile crystal contacts especially readily, opening the possibility of using these rigid multi-DARPin scaffolds to facilitate the crystallographic analysis of DARPin-target complexes that would not yield crystals otherwise. In the absence of its ligand, the interactions of DARPin *D12* are weak enough for constructs containing this DARPin to remain monomeric during expression and purification. Upon crystallization, the paratope-paratope interactions of this DARPin are strong enough to form prominent crystal contacts under a wide variety of conditions (Fig. 5). This DARPin may thus be added to the portfolio of well crystallizing proteins such as, e.g., T4 lysozyme^20^, apo-cytochrome B(562)RIL and several others^21^, yet with the additional advantage of being able to be fused to other proteins in a rigid manner and be part of binding complexes. DARPin-target complexes that fail to crystallize can be expanded by rigidly fused *D12* modules on either side of the DARPin of interest to provide additional crystal contacts. This approach enabled us to produce diffraction-quality crystals and thus understand the mode of action of two DARPins bound to their targets (Y. Wu *et al*., manuscript submitted), when all previous attempts to crystallize the DARPin-target complexes had failed. Since target-binding DARPins can now be rapidly generated from library selections^6^, and the capping repeats of each binder can then be replaced by a generic set of modular fusion constructs, this overall strategy can also be used to facilitate protein crystallization in general, and these fusions can be used as a next-generation crystallization chaperone.

The structures of *off7*:MBP_H10_*3G124*:GFP and *off7*:MBP_H11_*3G124*:GFP (Fig. 4) confirm that DD fusions are capable of crystallizing with two target proteins bound simultaneously. Modeling predicts that all nine DD constructs and 76 out of 81 DDD constructs should be able to bind two or three MBP-sized ligands simultaneously, respectively. The experimental structures elucidated in this study demonstrate this point: although all design steps were performed on models based on a single consensus DARPin not binding to any target (PDB ID: 2XEE,^22^), the internal repeats of the individual DARPin units in the fusion constructs could be readily replaced and indeed recognized the cognate target in the expected manner.

The propensity of DARPin *D12* to engage in crystal contacts is so large that, where the crystal geometry does not allow for its preferred interaction, alternative interaction geometries are observed. These are found as paratope-paratope interactions, interactions with the DARPin N- and C-caps, with connector modules or with the target protein of a second DARPin in the construct. Occasionally, the DARPin fusion constructs would rather distort the conformation of the connector module than forgo the formation of such favorable crystal contacts. Where we found deviations between design and experimental structures, the deviations can be explained either by crystal interactions and/or crystallization conditions.

The largest deviation was found for construct *D12*_H09_*D12* (Supplementary Fig. 4a), where the loop and first helix of the connector module (residues 141–156) were disordered in order to accommodate the second DARPin to engage in paratope-paratope type II interactions, Fig. 5b). In order to form an antiparallel dimer in which both *D12* moieties engage in this interaction, the second *D12* moiety had to rotate along its long axis.

In the *off7*_H09_*3G124* structure, residues 141–156 are clearly visible in the electron density map, but the angle between the last helix of the N-terminal DARPin domain and the H09 connector substantially differs from the design (Supplementary Fig. 4c). However, this construct crystallized at pH 4.6. DARPin repeats carry a conserved TPLH motif in the first helix of the N-cap^5, 23^, and protonation of the histidine residues in this motif reduces stability and weakens the binding affinity of the DARPin moiety, leading to the loss of MBP and GFP from the ternary complex. Nonetheless, independent binding data at neutral pH strongly indicate that the conformation in solution must be closely similar to the design^24^.

In summary, the strategy of rigid protein fusions, by embedded shared helices, has been found to be a versatile and modular approach to design a range of rigid proteins of predetermined dimension and orientation. It has greatly increased the utility of DARPins as crystallization chaperones by extending the search space for crystal formation through the dimensions of geometry, surface composition and orientation. This strategy should expand the toolbox for structure determination. However, potential applications of rigid DARPin-DARPin fusions go far beyond their utility as crystallization chaperones: these constructs can be used to organize supramolecular complexes of different geometries, and they can modulate the activity of target molecules not only by the direct effects of the DARPin moiety binding to its targets, but by bringing different targets together, forcing them into a specific relative orientation and modulating their conformation. The crystal structures presented in this article show the general validity of this strategy, and thus help the future design of supramolecular complexes based on DARPin-rigid helix scaffolds with various target proteins.

## Materials and Methods

### Design

PyMOL (www.pymol.org) was used for initial screening for non-clashing DARPin/helix/DARPin arrangements. InsightII (Accelrys, SanDiego) was used to stitch the fragments together into one continuous chain and to regularize the backbone at the splice sites. Rosetta *fixbb* ^17^ was used for sequence optimization, and Rosetta *relax* to confirm that the backbone conformation remained stable upon energy minimization.

### Protein expression, purification and characterization

Expression of DARPin-based fusion constructs and target proteins, including DD fusions, DDD fusions, MBP, and GFP were all carried out with plasmid pQE30ss (a pQE30-derived vector with double stop codon) containing an N-terminal MRGS-His_6_ tag, and produced in *E. coli* XL-1 Blue (see Supplementary Experimental Procedures for details). MBP and GFP were biotinylated at the avi tag *in vivo* by co-transfected plasmid pBirAcm in *E. coli* XL-1 Blue (Stratagene), according to the protocols of Avidity and QIAGEN.

Proteins used for crystallization were purified by immobilized metal-ion affinity chromatography (IMAC). The elution buffer was exchanged to HBS_150_ (10 mM HEPES-Na, pH 7.4, 150 mM NaCl) on PD-10 desalting columns (GE Healthcare) according to the manufacturer’s instructions. Size exclusion chromatography was performed for further purification of complexes of DD constructs with their targets (MBP or GFP) (see Supplementary Experimental Procedures for details). To qualitatively assess whether DARPin-DARPin fusion constructs retain binding to their target(s), all expressed and purified DD fusion constructs were individually tested for binding to their target(s) by ELISA (see Supplementary Experimental Procedures for details).

### Crystallization, data collection and processing

Crystallization was set up using a variety of screens available at the in-house protein crystallization center. The proteins were concentrated to 10–25 mg/ml and mixed with the mother liquor in a volume ratio of 1:1, 1:2 and 2:1 for each single condition. Grid screening optimization of crystallization conditions was done with sitting-drop crystallization plates from Hampton Research. Crystal growth took place at 20 °C. Crystals typically appeared within a week and grew to their maximum size within 2 to 3 weeks. Crystallization conditions are summarized in Supplementary Table 1. Initial screens revealed that some of the DD fusion variants crystallized readily over a wide pH range. After optimization with custom-made focal grid screens, the best crystals were used in X-ray diffraction experiments. Diffraction data were collected at the Swiss Light Source, and the structures were determined by molecular replacement (See Supplementary Experimental Procedures for details).

### Accession number

The atomic coordinates of DARPin *D12*, the DARPin–DARPin fusions and complexes with their cognate targets have been deposited in the PDB (PDB ID: 5LE2, 5LE3, 5LE4, 5LE6, 5LE7, 5LE8, 5LE9, 5LEA, 5LEB, 5LEC, 5LED, 5LEE, 5LEL, 5LEM, 5LW2).

## Electronic supplementary material

Supplementary Figures and Methods
