# Supplementary material for: Rigidly connected multispecific artificial binders with adjustable geometries
Source: Sci Rep. 2017 Sep 11;7:11217. doi: 10.1038/s41598-017-11472-x (PMC5593856; doi:10.1038/s41598-017-11472-x)
Supplement: Supplementary file 1 — Supplementary Figures and Methods [file 41598_2017_11472_MOESM1_ESM.pdf]

Supplementary Information for

Rigidly connected multispecific artificial binders with adjustable geometries

Yufan Wu[1,2,*], Alexander Batyuk[1,3,*], Annemarie Honegger[1,*], Fabian Brandl[1], Peer R. E. Mittl[1], and Andreas Plückthun[1]

[1]Department of Biochemistry, University of Zürich, Winterthurerstrasse 190, CH-8057 Zürich, Switzerland

[2]Present address: Paul Scherrer Institute, OFLC/106, 5232 Villigen PSI, Switzerland

[3]Present address: Linac Coherent Light Source, SLAC National Accelerator Laboratory, 2575 Sand Hill Road, Menlo Park, CA 94025, USA

[*] Y. Wu, A. Batyuk and A. Honegger contributed equally to this work.

Correspondence to Andreas Plückthun: plueckthun@bioc.uzh.ch

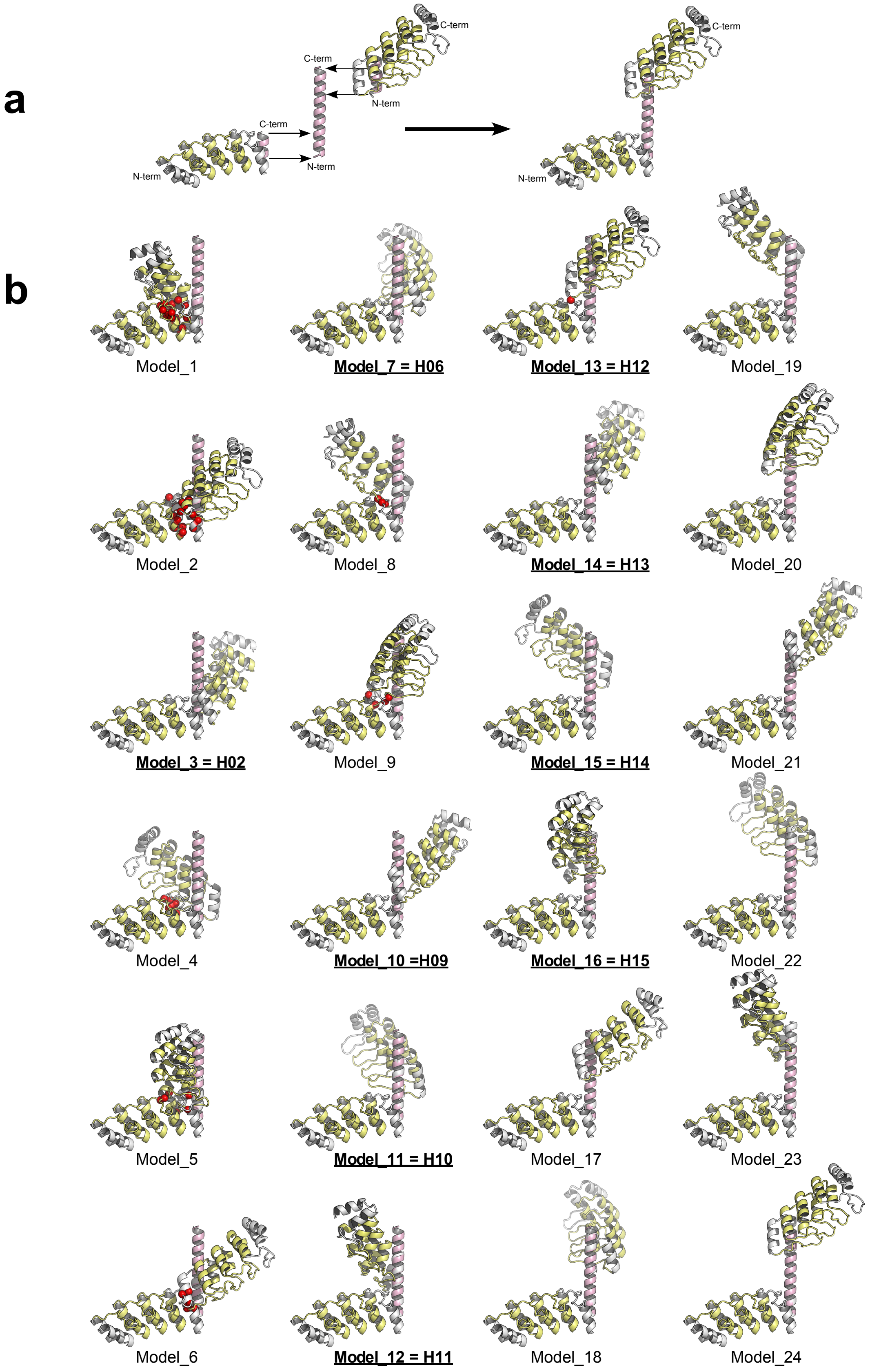
a
C-term
N-term
C-term
N-term
N-term
C-term
C-term
N-term
b
Model_1
Model_7 = H06
Model_13 = H12
Model_19
Model_2
Model_8
Model_14 = H13
Model_20
Model_3 = H02
Model_9
Model_15 = H14
Model_21
Model_4
Model_10 =H09
Model_16 = H15
Model_22
Model_5
Model_11 = H10
Model_17
Model_23
Model_6
Model_12 = H11
Model_18
Model_24

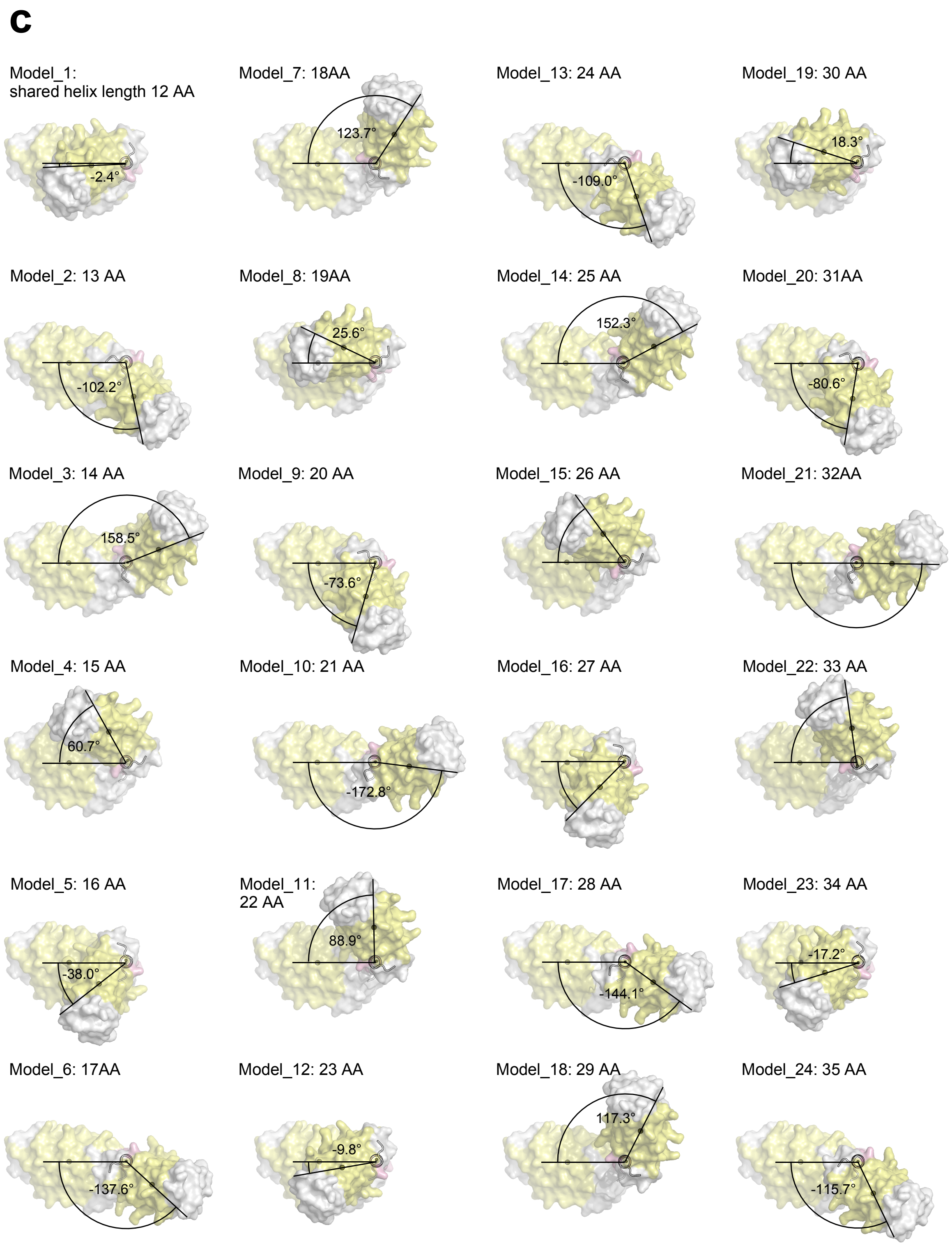

Supplementary Figure 1| Raw models of DD constructs, generated by a PyMOL script, available from the authors upon request**. (a)** A rigid fusion of a DARPin to a second DARPin is achieved through joining the C-terminal helix of the first DARPin to the N-terminal helix

of the second DARPin. A continuous helix is formed that is embedded in at least one of the two DARPins along its entire length. Different alignments of the two helices result in different relative orientations of the two DARPins. **(b)** Different fusions would result in clashes within the fused domains (depicted by red spheres). Underlined models were realized experimentally. The shared helix is oriented vertically in the image plane. (**c**) The relative orientation of the two domains is defined as the pseudo-torsion angle between the centers of gravity of the Cα positions of the two DARPins (residues 37-135, the three internal repeats). Views are along the shared helix.

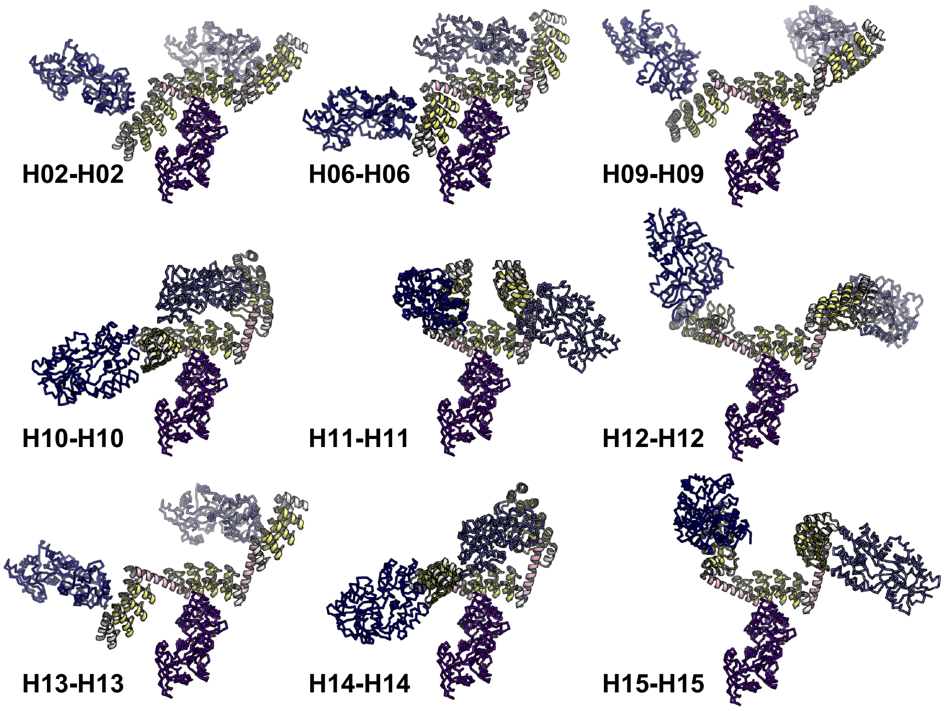

Supplementary Figure 2| Raw models of symmetric DDD constructs containing three modules of DARPin off7 in complex with MBP (PDB 1SVX), for illustrating orientations and distances. DARPin units are shown in pale yellow, shared helices in pink, MBP in dark blue and purple. All 81 DDD models were generated using a PyMOL script available from the authors upon request. To analyze the models for potential interference between the paratopes, the off7:MBP complex was superimposed on the three DARPin modules by a least-squares fit of the three internal repeat of the DARPin, and the models analyzed for clashes between the three MBP molecules. In "asymmetric" DDD constructs DDD_H11_H10, DDD_H11_H14, DDD_H12_H06 and DDD_H15_H14, MBP bound to DARPin modules 1 and 3 would clash (not shown), for all other DDD constructs three MBP molecules can bind simultaneously without clashing. Only the "symmetric" DDD constructs containing the same connector module between DARPin 1 and 2 and between DARPins 2 and 3 are shown.

If two paratopes are represented by the centers of gravity of the randomized residues, and the distance between the paratopes is defined as the distance between those two points, the distances between the paratopes of adjacent DARPins are: DD_H02, 42.1 Å; DD_H06,

40.9Å; DD_H09, 49.1 Å; DD_H10, 39.0 Å; DD_H11, 35.3Å; DD_H12 49.6 Å; DD_H13. 50.5Å; DD_H14, 39.4 Å; DD_H15, 45.5 Å.

The distance between the paratopes of DARPin 1 and 3 in the "symmetric" DDD constructs are: DDD_H02_H02, 78.2 Å ; DDD_H06_H06, 77.1 Å; DDD_H09_H09, 92.2 Å; DDD_H10_H10, 71.2Å; DDD_H11_H11, 38.9 Å; DDD_H12_H12, 88.2 Å; DDD_H13_H13, 96.9 Å; DDD_H14_H14, 67.9; DDD_H15_H15, 62.2 Å.

However, whether two ligands clash in a given construct depends more on the relative spatial orientation of the two paratopes than on their distance. The spacing and orientation of the paratopes can be further modified by altering the size of the DARPin moieties through insertion of additional, non-binding repeats between the connector modules and the internal repeats carrying the paratope.

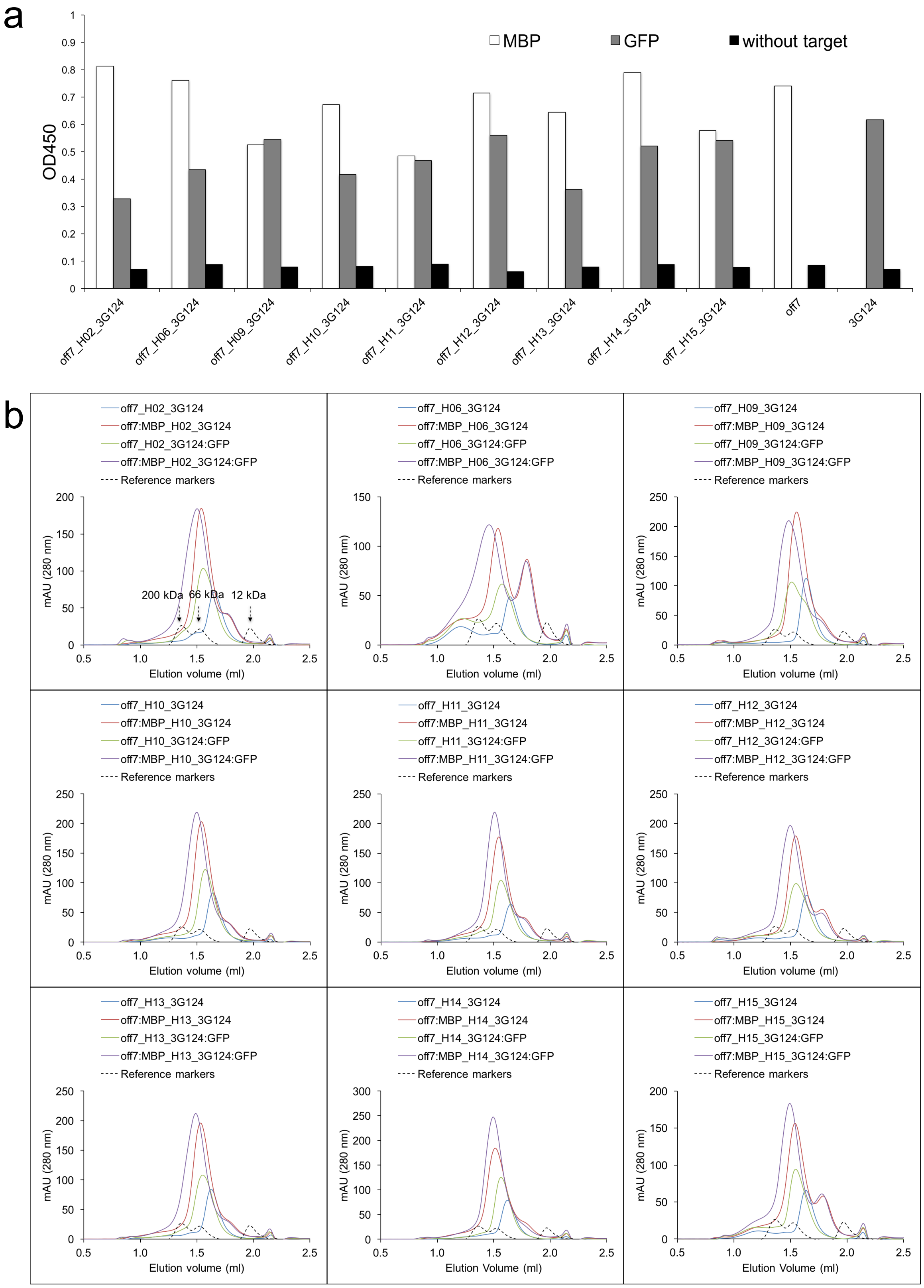

Supplementary Figure 3| Qualitative binding of DD fusions to targets, measured by ELISA. (a) Nine purified *off7*_Hxx_*3G124* fusions plus DARPin *off7* and *3G124* as positive controls were analyzed for binding to MBP and GFP. *White bars,* binding to immobilized

MBP; *gray bars,* binding to immobilized GFP; *black bars,* no target. **(b)** Size exclusion chromatography results of DD fusions with target(s). The elution volume and apparent molecular weight of the nine *off7*_Hxx_*3G124* fusion constructs is consistent with the molecular mass of the monomers. Clear shifts of the apparent molecular weights by 42 kDa, 29 kDa and 71 kDa are observed upon the binding of MBP alone, GFP alone and both MBP and GFP, respectively.

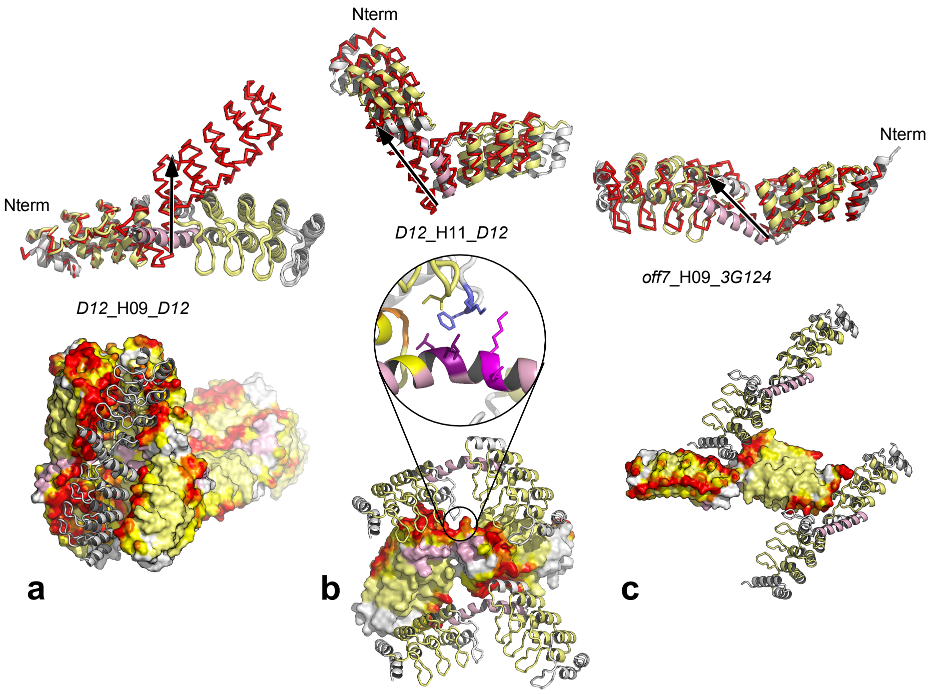

Supplementary Figure 4| Discrepancies between designed and experimental structures. Structures *D12*_H09_*D12*, *D12*_H11_*D12* and *off7*_H09_*3G124* showed a marked discrepancy between the design and the experimental structures found in the crystal. However, this is mainly an artefact introduced by crystal interactions and crystallization conditions, and may not reflect the predominant conformation in solution. *Top*: Model (red trace) and one chain of the structure (pastel cartoon) aligned by the internal repeats of the first DARPin to emphasize the differences. The structural deviation between the six chains within the asymmetric unit (a.u.) of *D12*_H09_*D12* is negligible (Supplementary Table 3). The helix axis in the model is indicated by an arrow. *Bottom*: Crystal contacts have been color-coded in the space-filling representation of the asymmetric unit: Atoms in direct Van-der-Waals contact (within 3.6 Å) with a different chain or with a symmetry-related molecule are colored red, solvent-excluding contacts orange (within 5.0 Å of another chain in the a.u. or a symmetry-related molecule). **(a)** In structure *D12*_H09_*D12*, the strong interaction between two *D12* paratopes favors a conformation in which both paratopes of the constructs can interact, even if this distorts the conformation of the connector module**.** **(b)** In structure *D12*_H11_*D12*, both the packing of the *D12* paratopes and direct side chain interactions with the shared helix of the connector module (blow-up) favor a kink in the helix. **(c)** The DD construct in *off7*_H09_*3G124* was crystallized at pH 4.6, destabilizing the construct and

resulting in the loss of both ligands (MBP and GFP). The low pH facilitated the unfolding of the C-cap-derived loop, disordered in this structure, and the dissociation of the shared helix from the last internal repeat of the first DARPin, allowing the N-terminal capping repeat of a symmetry mate to bind into the notch.

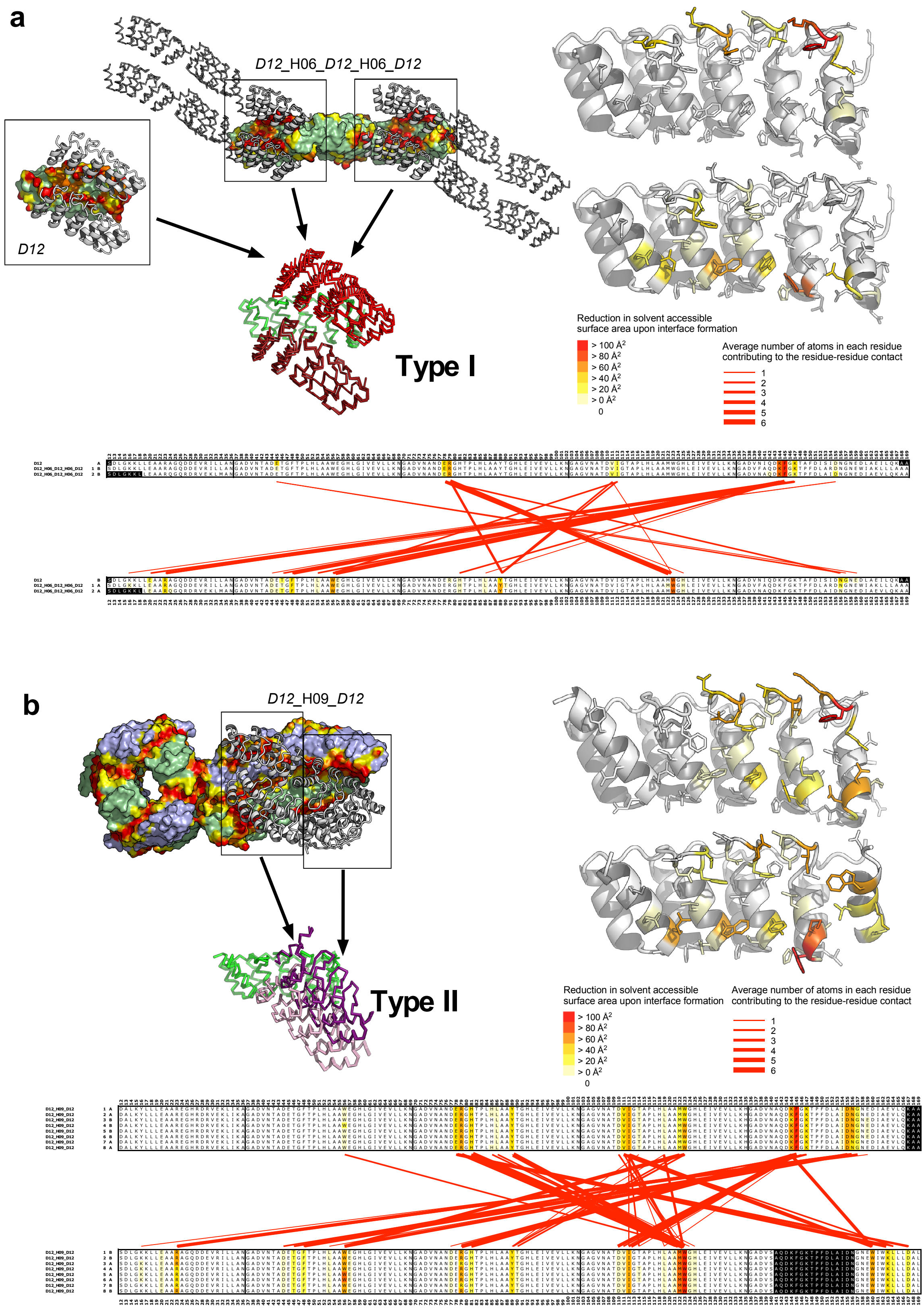
a
D12_H06_D12_H06_D12
D12
Type I
Reduction in solvent accessible surface area upon interface formation
> 100 Å2
> 80 Å2
> 60 Å2
> 40 Å2
> 20 Å2
> 0 Å2
0
Average number of atoms in each residue contributing to the residue-residue contact
1
2
3
4
5
6
b
D12_H09_D12
Type II
Reduction in solvent accessible surface area upon interface formation
> 100 Å2
> 80 Å2
> 60 Å2
> 40 Å2
> 20 Å2
> 0 Å2
0
Average number of atoms in each residue contributing to the residue-residue contact
1
2
3
4
5
6

c

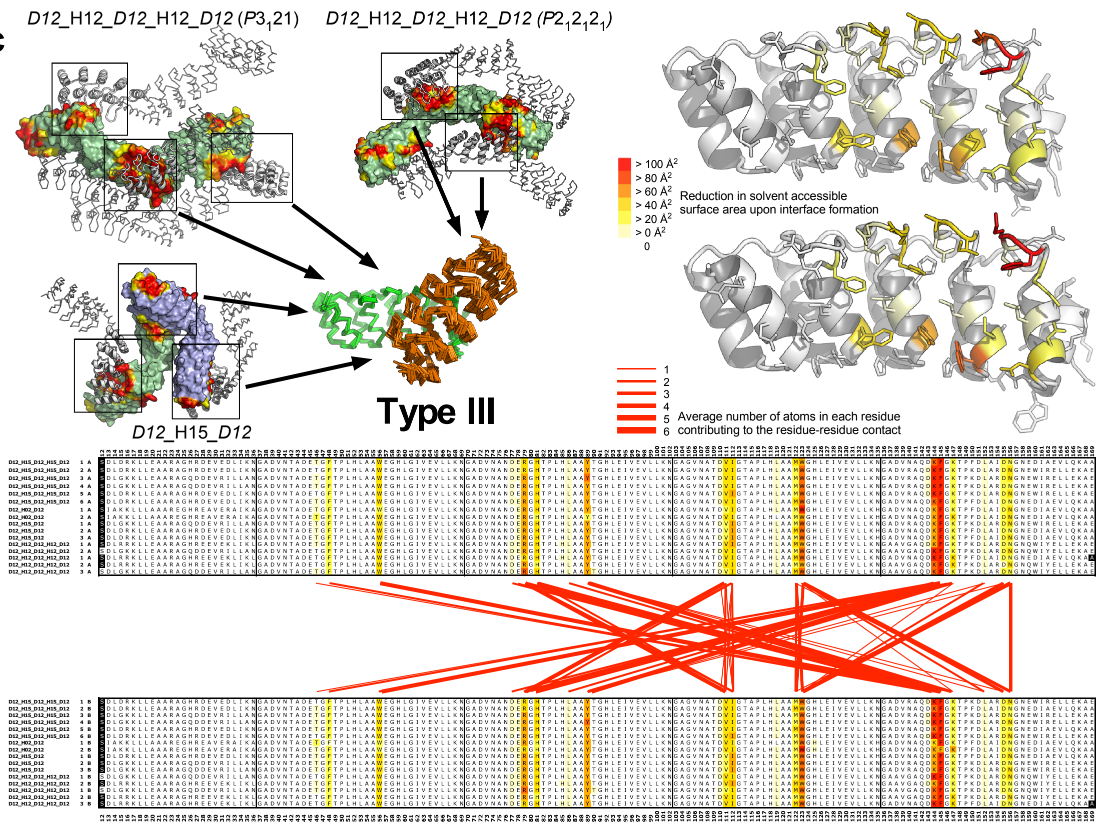
D12_H12_D12_H12_D12 (P3121)
D12_H12_D12_H12_D12 (P212121)
D12_H15_D12
Type III
> 100 Å2
> 80 Å2
> 60 Å2
> 40 Å2
> 20 Å2
> 0 Å2
0
Reduction in solvent accessible surface area upon interface formation
1
2
3
4
5
6
Average number of atoms in each residue contributing to the residue-residue contact

d

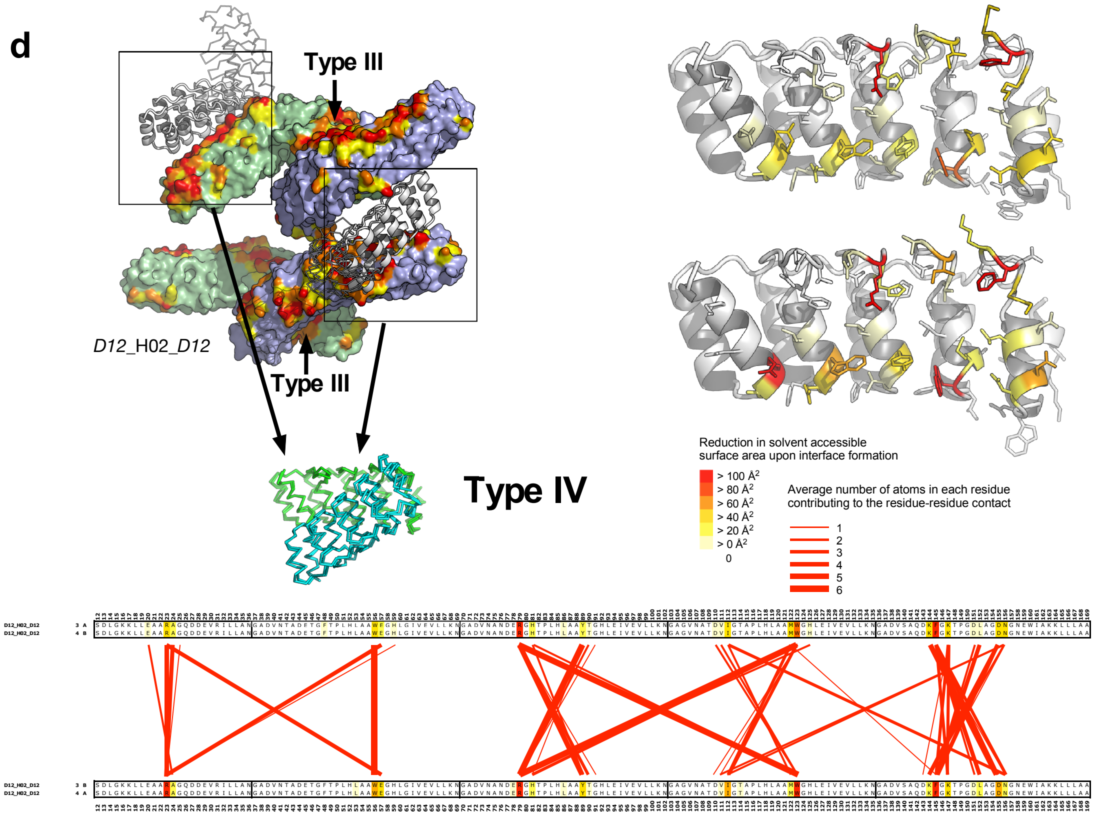
Type III
D12_H02_D12
Type III
Type IV
Reduction in solvent accessible surface area upon interface formation
> 100 Å2
> 80 Å2
> 60 Å2
> 40 Å2
> 20 Å2
> 0 Å2
0
Average number of atoms in each residue contributing to the residue-residue contact
1
2
3
4
5
6

e

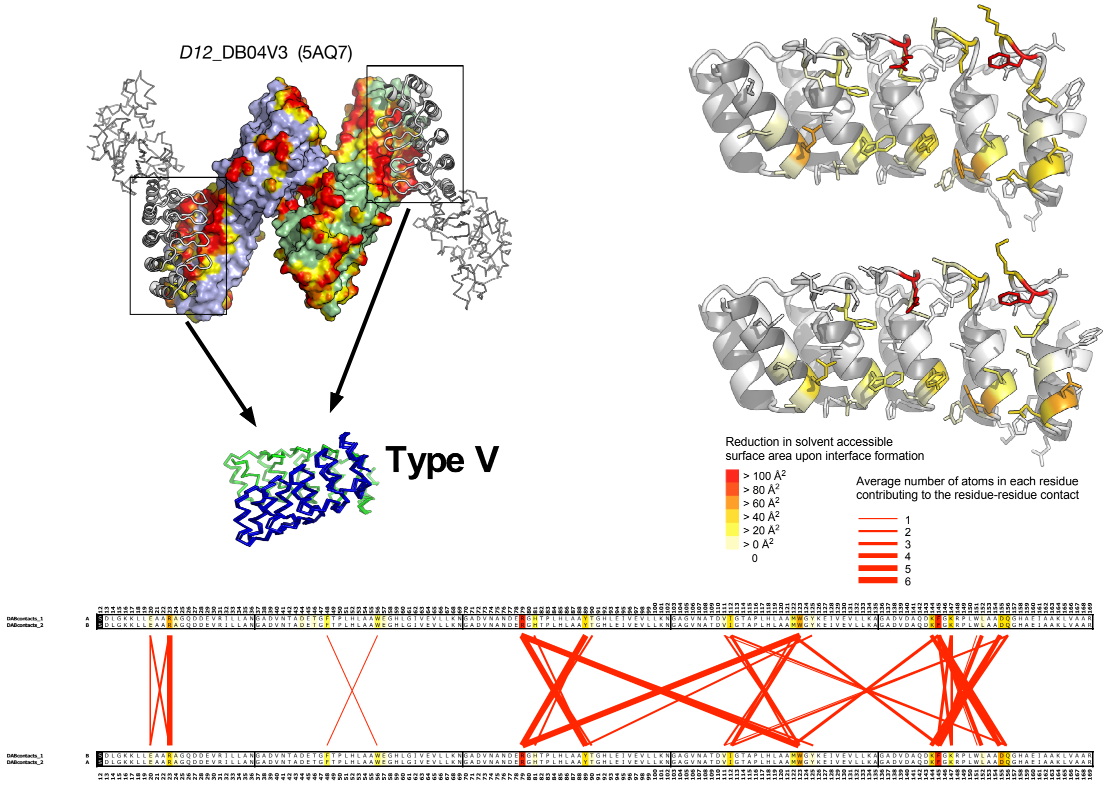

f

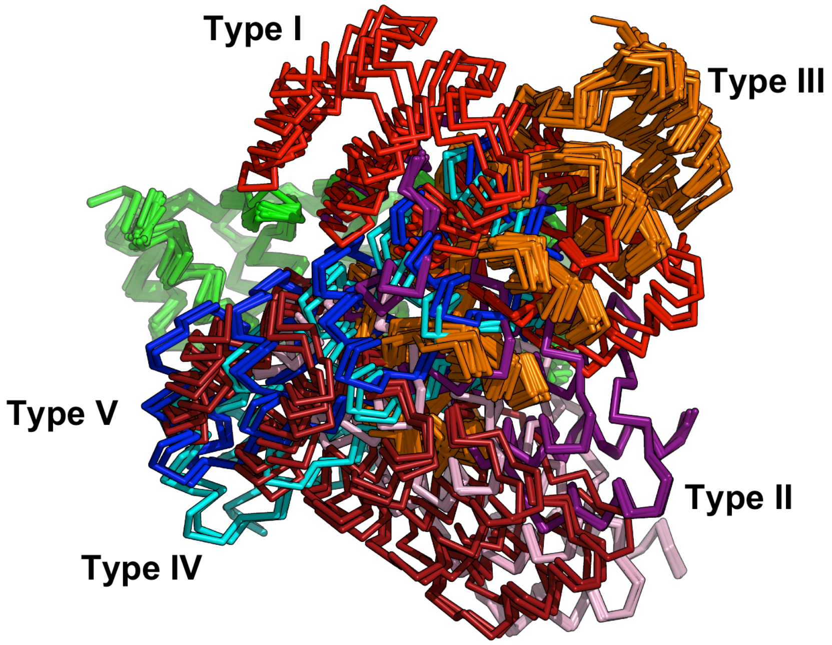

Supplementary Figure 5| *D12* paratope-paratope interactions as dominant crystal packing interactions in *D12*-based constructs. To analyze the paratope-paratope interactions, the two DARPin modules involved in the contact were copied into a new object

and 3D-aligned by either one or the other of the two DARPins, then clustered according to the different relative orientations of the DARPins. *Top left* in each panel: For the molecule(s) in the asymmetric unit, the solvent accessible surface is shown in pale green or pale blue, all crystal contacts are color-coded onto the surface: yellow for amino acids involved in the contact; orange, atoms directly involved in solvent excluding contacts; red, atoms involved in Van-der-Waals contacts. DARPin moieties in symmetry-related molecules that are involved in paratope-paratope interaction are shown as cartoon, the rest of the molecules as ribbon. *Top right*: For each pair of DARPin moieties involved in a paratope-paratope contact, the loss of solvent-accessible surface upon interaction for each residue was calculated using the program NACCESS and color-coded onto the structure. *bottom*: Sequences were color coded according to the loss of solvent accessible surface upon interaction for each residue, connecting lines represent the number of atom-atom contacts between the residues. Panels (**a)** to **(e**) illustrate interface types I-V according to Figure 5 in the main text, panel (**f**) shows a superposition of the different interface types.

Supplementary Table 1| Crystallization conditions, data collection and refinement details

	D12*_H02_*D12 (PDB ID 5LE3)	***D12*_H09_*D12*** (5LE6)	***D12*_H11_*D12*** (5LE4)	***D12*_H13_*D12*** (5LE7)
Data collection				
Space group	$P2_12_12$	$P6_122$	$P2_12_12_1$	$C2$
Molecules/AU	4	6	1	4
Unit cell parameters				
a, b, c (Å)	97.22, 108.79, 129.12	145.12, 145.12, 376.76	54.29, 57.08, 147.56	147.94, 75.25, 131.83
α, β, γ (°)	90, 90, 90	90, 90, 120	90, 90, 90	90, 96.935, 90
Observed reflections	229334 (22582)	8366503 (770629)	253695 (26472)	304462 (24870)
Unique reflections	17776 (1723)	215348 (21252)	19812 (1918)	83045 (7880)
Multiplicity	12.9 (12.9)	38.9 (36.2)	12.8 (13.6)	3.7 (3.2)
Completeness (%)	99 (99)	100 (100)	100 (100)	99 (95)
Mean I/sigma(I)	7.96 (0.38)	19.99 (1.10)	14.16 (0.70)	7.36 (0.44)
CC(1/2)	0.999 (0.387)	0.999 (0.593)	0.999 (0.446)	0.998 (0.123)
Wilson B-factor	155.75	28.91	67.90	56.93
Refinement				
Resolution range (Å)	48.21 - 3.5 (3.625 - 3.5)	48.26 - 1.8 (1.864 - 1.8)	39.34 - 2.35 (2.434 - 2.35)	45.56 - 2.104 (2.179 - 2.104)
*R*work (%)	29.89 (53.47)	19.22 (34.15)	23.24 (51.33)	22.54 (46.93)
*R*free (%)	35.15 (56.19)	22.64 (36.60)	27.17 (56.00)	25.79 (49.09)
Ordered water molecules	0	1758	0	152
Protein atoms	9100	13367	2375	9545
rmsd of bond lengths	0.001	0.006	0.002	0.003
rmsd of bond angles	0.47	0.73	0.47	0.49
Average *B*-factor	188.78	36.51	102.09	68.07
Ramachandran plot (%)				
Favored	93	99	96	98
Allowed	6.7	0.8	4.2	1.6
Outliers	0	0	0	0
Crystallization conditions	PEG 3350 15.9% w/v<br>Sodium fluoride 0.2 M<br>Bis Tris propane 0.1 M<br>pH 6.3	PEG 3350 17% w/v<br>Na_2SO_4 0.2 M	PEG 3350 18% w/v<br>KSCN 0.15 M<br>Tris(HOAc) 0.1 M<br>pH 7.5	PEG 3350 25% w/v<br>$(NH_4)_2SO_4$ 0.2 M<br>Bis-Tris 0.1 M<br>pH 5.5

Values in parentheses refer to the outermost resolution shell.

	D12*_H15_*D12 **(5LE8)**	***off7*_H09_*3G124*** **(5LE9)**	***off7*:MBP_H10_*3G124*:GFP** **(5LEL)**	***off7*:MBP_H11_*3G124*:GFP** **(5LEM)**
Data collection				
Space group	*P*1	$P4_12_12$	*C*2	$P2_12_12$
Molecules/AU	2	1	9 (3 complexes)	3 (1 complex)
Unit cell parameters				
a, b, c (Å)	54.76, 73.54, 77.98	71.98, 71.98, 128.19	235, 103.14, 156.84	69.43, 169.95, 95.97
α, β, γ (°)	96.032, 103.431, 89.515	90, 90, 90	90, 131.355, 90	90, 90, 90
Observed reflections	381407 (37327)	734077 (69711)	342770 (34447)	154345 (15718)
Unique reflections	109030 (9463)	29498 (2843)	51171 (5070)	23885 (2129)
Multiplicity	3.5 (3.5)	24.9 (24.3)	6.7 (6.8)	6.5 (6.7)
Completeness (%)	95 (94)	99 (99)	100 (99)	99 (99)
Mean I/sigma(I)	8.62 (0.38)	13.26 (0.23)	5.81 (0.18)	8.65 (0.18)
CC(1/2)	0.997 (0.15)	0.999 (0.336)	0.993 (0.0529)	0.998 (0.0502)
Wilson B-factor	38.69	40.56	85.18	127.80
Refinement				
Resolution range (Å)	41.9 - 1.78 (1.844 - 1.78)	39.86 - 1.85 (1.916 - 1.85)	44.52 - 3.1 (3.211 - 3.1)	48.78 - 2.98 (3.087 - 2.98)
*R*work (%)	18.05 (41.71)	22.57 (50.53)	25.83 (40.07)	24.47 (45.95)
*R*free (%)	20.52 (41.96)	24.50 (47.79)	30.60 (38.52)	29.47 (47.40)
Ordered water molecules	532	124	0	1
Protein atoms	4875	2410	20980	7063
rmsd of bond lengths	0.005	0.002	0.002	0.002
rmsd of bond angles	0.72	0.43	0.50	0.44
Average *B*-factor	48.45	53.49	108.50	150.97
Ramachandran plot (%)				
Favored	100	98	97	93
Allowed	0	1.9	2.8	6.6
Outliers	0	0	0	0.11
Crystallization conditions	$(NH_4)2_SO_4$ 1.5 M<br>Tris(HOAc) 0.1 M<br>pH 8.5	MPD 30% v/v<br>NaCl 0.2 M<br>Na acetate trihydrate 0.1 M<br>pH 4.6	PEG 3350 23.2% w/v<br>Sodium acetate 0.2 M<br>Bis Tris propane 0.1 M<br>pH 8.0	PEG 6000 20% w/v<br>TAPS 0.02 M<br>pH 9.0

Values in parentheses refer to the outermost resolution shell.

	***off7*_H12_*3G124* (5LEA)**	***D12*_H06_*D12*_H06_*D12* (5LEB)**	***D12*_H12_*D12*_H12_*D12* (5LEC)**	***D12*_H12_*D12*_H12_*D12* (5LED)**
Data collection				
Space group	$P2_1$	$C2$	$P2_12_12_1$	$P2_1$
Molecules/AU	1	1	1	1
Unit cell parameters				
a, b, c (Å)	62.41, 46.4, 63.08	129.12, 29.47, 146.87	95.15, 104.59, 57	60.23, 47.55, 82.48
α, β, γ (°)	90, 111.098, 90	90, 107.466, 90	90, 90, 90	90, 92.042, 90
Observed reflections	56028 (5600)	161058 (16520)	261340 (26341)	88985 (8172)
Unique reflections	13295 (1309)	24249 (2378)	20168 (1968)	14376 (1392)
Multiplicity	4.2 (4.1)	6.6 (6.9)	13.0 (13.4)	6.2 (5.9)
Completeness (%)	99 (97.2)	100 (100)	100 (100)	98 (97)
Mean I/sigma(I)	9.4 (1.2)	18.17 (0.99)	8.63 (0.51)	5.16 (0.54)
CC(1/2)	0.997 (0.571)	1 (0.468)	0.997 (0.349)	0.993 (0.233)
Wilson B-factor	53.69	57.25	73.08	62.79
Refinement				
Resolution range (Å)	36.29 - 2.4 (2.486 - 2.4)	35.02 - 2.3 (2.382 - 2.3)	43.31 - 2.506 (2.596 - 2.506)	41.21 - 2.6 (2.693 - 2.6)
*R*work (%)	23.60 (33.60)	21.13 (40.27)	23.28 (43.17)	25.52 (39.18)
*R*free (%)	28.43 (36.90)	24.12 (43.52)	26.56 (46.19)	28.98 (41.34)
Ordered water molecules	17	26	9	0
Protein atoms	2391	3469	3589	3443
rmsd of bond lengths	0.002	0.002	0.002	0.002
rmsd of bond angles	0.46	0.43	0.44	0.43
Average *B*-factor	81.01	68.90	90.24	88.54
Ramachandran plot (%)				
Favored	96	98	97	95
Allowed	4.1	1.7	2.8	4.6
Outliers	0	0	0	0
Crystallization conditions	MPD 32.3% v/v<br>NaCl 0.2 M<br>Sodium acetate trihydrate 0.1 M<br>pH 5.0	PEG 8000 10.0% w/v<br>PEG 1000 10.0% w/v<br>Sodium acetate 0.3 M<br>Sodium cacodylate 0.1 M<br>pH 6.5	PEG 3350 20% w/v<br>Citric acid 0.04 M<br>Bis Tris propane 0.06 M<br>pH 6.4	PEG 4000 20% w/v<br>Tris(HOAc) 0.1 M<br>$CdCl_2$ 0.005 M<br>pH 8.5

Values in parentheses refer to the outermost resolution shell.

	*D12*_H12_*D12*_H12_*D12* (5LEE)	*D12*_H15_*D12*_H15_*D12* (5LE2)	*D12* (5LW2)
Data collection			
Space group	$P3_221$	$P2_1$	*C*2
Molecules/AU	1	2	1
Unit cell parameters			
a, b, c (Å)	69.26, 69.26, 230.49	92.97, 53.79, 144	96.13, 28.00, 52.73
α, β, γ (°)	90, 90, 120	90, 107.108, 90	90, 104.533, 90
Observed reflections	502120 (49599)	351368 (33746)	51033 (4939)
Unique reflections	25927 (1779)	53205 (4809)	14006 (1382)
Multiplicity	19.4 (19.9)	6.6 (6.6)	3.6 (3.6)
Completeness (%)	97 (99)	98 (95)	99 (100)
Mean I/sigma(I)	18.38 (0.08)	6.13 (0.13)	7.41 (0.90)
CC(1/2)	0.999 (0.393)	0.997 (0.163)	0.996 (0.529)
Wilson B-factor	94.84	67.68	27.65
Refinement			
Resolution range (Å)	41.55 - 2.401 (2.487 - 2.401)	48 - 2.4 (2.486 - 2.4)	39.7 - 1.75 (1.813 - 1.75)
*R*work (%)	24.93 (61.55)	24.76 (51.47)	22.04 (40.49)
*R*free (%)	27.40 (55.87)	28.09 (51.01)	26.09 (51.76)
Ordered water molecules	0	51	93
Protein atoms	3584	7294	2314
rmsd of bond lengths	0.002	0.003	0.003
rmsd of bond angles	0.43	0.47	0.452
Average *B*-factor	136.03	82.17	43.48
Ramachandran plot (%)			
Favored	96	96	98.7
Allowed	4	3.6	1.3
Outliers	0	0	0
Crystallization conditions	PEG 3350 20% w/v<br>Sodium citrate 0.2 M<br>Bis Tris propane 0.1 M<br>pH 8.5	PEG 8000 10% w/v<br>PEG 1000 10 % w/v<br>KSCN 0.2 M<br>Tris (HOAc) 0.1 M<br>pH 7.5	PEG 1000 10% w/v<br>PEG 8000 10% w/v<br>Ca-acetate 0.2 M<br>Tris(HOAc) 0.1 M<br>pH 7.5

Values in parentheses refer to the outermost resolution shell.

Supplemental Table 2: Comparison of Structures to Models

Structure	chain	Model	angle predicted	angle D1/D2	angle D2/D3	rmsd all	# of atoms	D1-connector-D2-connector-D3	# of atoms	D1-connector-D2 rmsd	# of atoms	D2-connector-D3 rmsd	# of atoms	Darpin 1 rmsd	# of atoms	connector 1 rmsd	# of atoms	Darpin 2 rmsd	# of atoms	connector2 rmsd	# of atoms	DARPin 3 rmsd	# of atoms
D12_H02_D12	A	H02	158.1	153.6		1.81	300			1.49	247			0.69	99	0.98	49	0.83	99				
D12_H02_D12	B	H02	158.1	162.9		1.29	300			1.20	247			0.81	99	0.84	49	0.78	99				
D12_H02_D12	C	H02	158.1	163.3		1.21	300			1.17	247			0.82	99	0.92	49	0.78	99				
D12_H02_D12	D	H02	158.1	159.6		1.25	300			1.18	247			0.81	99	0.95	49	0.82	99				
D12_H09_D12	A	H09	180.0	172.8		7.01	291			7.03	238			0.71	99	3.10	40	0.56	99				
D12_H09_D12	B	H09	180.0	172.7		7.03	291			7.04	238			0.73	99	3.13	40	0.57	99				
D12_H09_D12	C	H09	180.0	172.4		7.02	291			7.04	238			0.72	99	3.11	40	0.56	99				
D12_H09_D12	D	H09	180.0	172.2		7.00	291			7.03	238			0.73	99	3.15	40	0.54	99				
D12_H09_D12	E	H09	180.0	172.3		7.00	291			7.03	238			0.72	99	3.13	40	0.54	99				
D12_H09_D12	F	H09	180.0	172.2		6.98	291			7.01	238			0.70	99	3.04	40	0.55	99				
D12_H11_D12	A	H11	-19.7	-24.6		6.38	309			5.98	256			0.57	99	3.35	58	0.68	99				
D12_H13_D12	A	H13	141.0	147.7		1.32	311			1.18	258			0.62	99	0.64	60	0.59	99				
D12_H13_D12	B	H13	141.0	138.7		0.83	311			0.79	258			0.62	99	0.47	60	0.57	99				
D12_H13_D12	C	H13	141.0	138.9		0.71	311			0.71	258			0.64	99	0.46	60	0.61	99				
D12_H13_D12	D	H13	141.0	149.6		1.46	311			1.28	258			0.58	99	0.66	60	0.57	99				
D12_H15_D12	A	H15	60.5	63.9		1.84	313			1.76	260			0.57	99	1.03	62	0.57	99				
D12_H15_D12	B	H15	60.5	64.8		2.11	313			1.98	260			0.57	99	1.12	62	0.59	99				
off7_H09_3G124	A	H09	180.0	127.5		6.04	307			5.72	254			0.50	99	4.87	56	0.46	99				
off7:MBP_H09_3G124:GFP	A	H10	82.2	112.6		4.03	308			3.64	255			0.66	99	1.92	57	0.58	99				
off7:MBP_H09_3G124:GFP	D	H10	82.2	113.4		3.99	308			3.59	255			0.59	99	1.96	57	0.53	99				
off7:MBP_H09_3G124:GFP	G	H10	82.2	113.2		4.29	308			3.93	255			0.67	99	2.02	57	0.69	99				
off7:MBP_H11_3G124:GFP	A	H11	-19.7	-17.6		1.74	309			1.51	256			0.54	99	0.71	58	0.66	99				
off7_H12_3G124	A	H12	-118.8	-113.6		1.01	310			0.91	257			0.46	99	0.63	59	0.69	99				
D12_H06_D12_H06_D12	A	H06_H06	-119.7	-133.5	-140.2	4.07	456	3.51	403	2.36	251	2.86	251	0.59	99	1.04	53	0.48	99	1.34	53	0.44	99
D12_H12_D12_H12_D12 ($P2_12_12_1$)	A	H12_H12	-118.8	-93.6	-113.0	4.17	468	3.63	415	2.47	257	1.06	257	0.67	99	1.22	59	0.47	99	0.89	59	0.44	99
D12_H12_D12_H12_D12 ($P2_1$)	A	H12_H12	-118.8	-109.6	-111.6	1.51	451	1.41	415	0.94	257	0.93	257	0.59	99	0.69	59	0.47	99	1.01	59	0.57	99
D12_H12_D12_H12_D12 ($P3_221$)	A	H12_H12	-118.8	-101.0	-103.1	2.83	468	2.52	415	1.84	257	1.42	257	0.65	99	1.08	59	0.56	99	0.95	59	0.79	99
D12_H15_D12_H15_D12	A	H15_H15	60.5	69.6	66.9	5.48	474	4.91	421	2.40	260	1.83	260	0.65	99	1.30	62	0.49	99	0.85	62	0.52	99
D12_H15_D12_H15_D12	B	H15_H15	60.5	69.6	64.3	4.35	474	3.73	421	1.62	260	1.74	260	0.73	99	0.76	62	0.49	99	0.76	62	0.52	99

Root-mean-square deviations between the Calpha atoms of structure and model were calculated using the PyMOL fit command, and interdomain pseudo-torsion angles.

Values indicative of a significant deviation from the design are colored in red.

Supplementary Table 3: Comparison between multiple molecules within the same structure

Structure	chain	chain	angle D1/D2	angle D1/D2	angle D2/D3	angle D2/D3	rmsd all	# of atoms	D1-connector-D2-connector-D3	# of atoms	D1-connector-D2 rmsd	# of atoms	D2-connector-D3 rmsd	# of atoms	Darpin 1 rmsd	# of atoms	connector 1 rmsd	# of atoms	Darpin 2 rmsd	# of atoms	connector2 rmsd	# of atoms	DARPin 3 rmsd	# of atoms
**D*12*_H02_D*12***	A	B	153.6	162.9			2.3	300			1.9	247			0.7	99	0.9	49	0.5	99				
**D*12*_H02_D*12***	A	C	153.6	163.3			1.7	300			1.4	247			0.6	99	0.8	49	0.5	99				
**D*12*_H02_D*12***	A	D	153.6	159.6			1.7	300			1.4	247			0.6	99	0.8	49	0.5	99				
**D*12*_H02_D*12***	B	C	162.9	163.3			1.0	300			1.0	247			0.7	99	0.9	49	0.5	99				
**D*12*_H02_D*12***	B	D	162.9	159.6			1.0	300			0.9	247			0.8	99	0.8	49	0.5	99				
**D*12*_H02_D*12***	C	D	163.3	159.6			0.6	300			0.5	247			0.5	99	0.5	49	0.4	99				
D12*_H09_*D12	A	B	172.8	172.7			0.3	291			0.2	238			0.2	99	0.4	40	0.1	99				
D12*_H09_*D12	A	C	172.8	172.4			0.2	291			0.2	238			0.1	99	0.4	40	0.1	99				
D12*_H09_*D12	A	D	172.8	172.2			0.2	291			0.2	238			0.1	99	0.4	40	0.2	99				
D12*_H09_*D12	A	E	172.8	172.3			0.2	291			0.2	238			0.1	99	0.4	40	0.1	99				
D12*_H09_*D12	A	F	172.8	172.2			0.2	291			0.2	238			0.1	99	0.3	40	0.2	99				
D12*_H09_*D12	B	C	172.7	172.4			0.1	291			0.1	238			0.1	99	0.1	40	0.1	99				
D12*_H09_*D12	B	D	172.7	172.2			0.3	291			0.2	238			0.1	99	0.3	40	0.2	99				
D12*_H09_*D12	B	E	172.7	172.3			0.3	291			0.2	238			0.1	99	0.4	40	0.1	99				
D12*_H09_*D12	B	F	172.7	172.2			0.3	291			0.2	238			0.1	99	0.3	40	0.2	99				
D12*_H09_*D12	C	D	172.4	172.2			0.2	291			0.2	238			0.1	99	0.3	40	0.2	99				
D12*_H09_*D12	C	E	172.4	172.3			0.2	291			0.2	238			0.1	99	0.4	40	0.1	99				
D12*_H09_*D12	C	F	172.4	172.2			0.2	291			0.2	238			0.1	99	0.2	40	0.2	99				
D12*_H09_*D12	D	E	172.2	172.3			0.1	291			0.1	238			0.1	99	0.2	40	0.1	99				
D12*_H09_*D12	D	F	172.2	172.2			0.2	291			0.2	238			0.1	99	0.3	40	0.1	99				
D12*_H09_*D12	E	F	172.3	172.2			0.2	291			0.2	238			0.1	99	0.3	40	0.1	99				
***off7:MBP*_H09_*3G124*:GFP**	A	D	112.6	113.4			0.6	308			0.5	255			0.3	99	0.3	57	0.3	99				
***off7:MBP*_H09_*3G124*:GFP**	A	G	112.6	113.2			1.1	308			1.1	255			0.3	99	0.9	57	0.4	99				
***off7:MBP*_H09_*3G124*:GFP**	D	G	113.4	113.2			1.3	308			1.2	255			0.3	99	1.0	57	0.4	99				
D12*_H13_*D12	A	B	147.7	138.7			1.6	311			1.4	258			0.3	99	0.6	60	0.2	99				
D12*_H13_*D12	A	C	147.7	138.9			1.4	311			1.2	258			0.3	99	0.5	60	0.3	99				
D12*_H13_*D12	A	D	147.7	149.6			0.3	311			0.3	258			0.2	99	0.2	60	0.2	99				
D12*_H13_*D12	B	C	138.7	138.9			0.4	311			0.4	258			0.1	99	0.2	60	0.2	99				
D12*_H13_*D12	B	D	138.7	149.6			1.8	311			1.5	258			0.2	99	0.7	60	0.3	99				
D12*_H13_*D12	C	D	138.9	149.6			1.5	311			1.4	258			0.3	99	0.6	60	0.3	99				
D12*_H15_*D12	A	B	63.9	64.8			0.4	313			0.3	260			0.1	99	0.1	62	0.1	99				
D12*_H15_*D12*_H15_*D12	A	B	69.6	69.6	66.9	64.3	2.1	474	2.1	421	1.7	260	0.35	260	0.3	99	1.4	62	0.1	99	0.3	62	0.2	99

Supplementary Table 4| Comparison between multiple structures containing the same connector modules

Connector	structure	chain	structure	chain	angle D1/D2	angle D1/D2	angle D2/D3	angle D2/D3	rmsd all	# of atoms	D1-connector-D2-connector-D3	# of atoms	D1-connector-D2 rmsd	# of atoms	D2-connector-D3	# of atoms	Darpin 1 rmsd	# of atoms	connector 1 rmsd	# of atoms	Darpin 2 rmsd	# of atoms	connector2 rmsd	# of atoms	DARPin 3 rmsd	# of atoms
H09	**D12_H09_D12**	A	**off7_H09_3G124**	A	172.8	127.5			6.36	291			5.88	238			0.534	99	1.875	40	0.523	99				
H09	**D12_H09_D12**	B	**off7_H09_3G124**	A	172.7	127.5			6.39	291			5.90	238			0.576	99	1.843	40	0.53	99				
H09	**D12_H09_D12**	C	**off7_H09_3G124**	A	172.4	127.5			6.35	291			5.87	238			0.557	99	1.828	40	0.527	99				
H09	**D12_H09_D12**	D	**off7_H09_3G124**	A	172.2	127.5			6.31	291			5.83	238			0.551	99	1.882	40	0.525	99				
H09	**D12_H09_D12**	E	**off7_H09_3G124**	A	172.3	127.5			6.31	291			5.83	238			0.541	99	1.842	40	0.5	99				
H09	**D12_H09_D12**	F	**off7_H09_3G124**	A	172.2	127.5			6.30	291			5.83	238			0.525	99	1.869	40	0.518	99				
H11	**D12_H11_D12**	A	**off7:MBP_H11_3G124:GFP**	A	-24.6	-17.6			5.19	309			4.96	256			0.50	99	3.126	58	0.767	99				
H12, H12_H12	**off7_H12_3G124**	A	**D12_H12_D12_H12_D12 ($P2_12_12_1$)**	A	-113.6	-93.6							2.25	257			0.482	99	1.176	59	0.545	99				
H12, H12_H12	**off7_H12_3G124**	A	**D12_H12_D12_H12_D12 ($P2_12_12_1$)**	A	-113.6	-113.0							1.14	257			0.721	99	0.893	59	0.63	99				
H12, H12_H12	**off7_H12_3G124**	A	**D12_H12_D12_H12_D12 ($P2_1$)**	A	-113.6	-109.6							0.89	257			0.451	99	0.658	59	0.433	99				
H12, H12_H12	**off7_H12_3G124**	A	**D12_H12_D12_H12_D12 ($P2_1$)**	A	-113.6	-111.6							1.08	257			0.60	99	0.985	59	0.691	99				
H12, H12_H12	**off7_H12_3G124**	A	**D12_H12_D12_H12_D12 ($P3_221$)**	A	-113.6	-101.0							1.74	257			0.52	99	1.046	59	0.477	99				
H12, H12_H12	**off7_H12_3G124**	A	**D12_H12_D12_H12_D12 ($P3_221$)**	A	-113.6	-103.1							1.33	257			0.68	99	0.92	59	0.787	99				
H12_H12	**D12_H12_D12_H12_D12 ($P2_12_12_1$)**	A	**D12_H12_D12_H12_D12 ($P2_1$)**	A	-93.6	-109.6	-113.0	-111.6	2.89	451	2.528	415	1.83	257	0.846	257	0.368	99	0.92	59	0.438	99	0.508	59	0.437	99
H12_H12	**D12_H12_D12_H12_D12 ($P2_12_12_1$)**	A	**D12_H12_D12_H12_D12 ($P3_221$)**	A	-93.6	-101.0	-113.0	-103.1	2.34	468	2.044	415	1.49	257	0.911	257	0.436	99	0.728	59	0.377	99	0.473	59	0.619	99
H12_H12	**D12_H12_D12_H12_D12 ($P2_1$)**	A	**D12_H12_D12_H12_D12 ($P3_221$)**	A	-109.6	-101.0	-111.6	-103.1	1.73	451	1.616	415	1.18	257	1.194	257	0.313	99	0.673	59	0.406	99	0.50	59	0.531	99

Values indicative of a significant deviation between two structures containing the same connector module are colored in red.

Supplementary Methods

Expression and purification of DARPin based fusion proteins

DARPin-based fusion proteins (DD and DDD constructs) were expressed from the plasmid pQE30ss (vector pQE30 with double stop codon) in the *E. coli* strain XL1-blue. The fusion proteins contain an N-terminal MRGS-His_6 tag. An overnight culture of each variant was prepared as follows: 50 mL 2xYT medium (100 µg/l ampicillin, 1% w/v glucose) was inoculated with a single colony cultured on LB plates (100 µg/l ampicillin, 1% w/v glucose, 1.5% agar). The overnight culture (225 rpm, 37 °C) typically reached an OD_{600} ~ 4.5. The seed cultures were then diluted to a final OD_{600} = 0.1 into 1 L of 2xYT medium (containing 100 mg/l ampicillin, 1% w/v glucose) in a 5-liter baffled flask. Expression cultures were placed in a shaker (25 mm radius, 108 rpm, 30 °C) until induction with IPTG (0.5 mM final concentration) at OD_{600} ~ 0.6. Induced cultures were then allowed to shake for 18 hours at 30 °C. Overnight expression cultures were harvested by centrifugation (5,000 *g*, 4°C, 15 min).

Cell pellets from 1-liter cultures were resuspended in 30 ml TBS_{400} buffer (50 mM Tris HCl pH 7.4, 400 mM NaCl) containing one EDTA-free protease inhibitor tablet (Roche), DNAse (10 µg), and $MgCl_2$ (10 mM final concentration), then homogenized with a Digitana YellowLine mixer and ruptured by one passage through a TS 1.1 Constant Cell Disrupter System at 30,000 psi. A subsequent sonication step (duty cycle 50%, intensity 5, 1-2 x 30 s pulses) was applied to the crude lysate for additional cell rupture and shearing of DNA. The lysate was clarified by centrifugation in SS34 tubes (30 min at 28,000 g, 4 °C) to give a final volume of ~30 ml. The supernatant was then filtered (0.22 µm Millex GP, Millipore) with a sterile syringe before adjusting the pH to ~ 8.0 with 0.5 M NaOH.

Immobilized metal-ion affinity chromatography (IMAC) on Ni^{2+}-nitrilotriacetic acid (Ni-NTA) resin was used for protein purification at room temperature. Four ml of 50% slurry of Ni-NTA Superflow (Qiagen) was applied to a 15 ml fitted chromatography column (Bio-Rad). Before application of the clarified supernatant, the column was washed with 5 column volumes (CV) of UHP water, and then equilibrated with 10 CV of TBS-W buffer (50 mM Tris HCl pH 7.4, 400 mM NaCl, 20 mM imidazole, 10% glycerol). After applying the clarified lysate, a washing step with TBS-W (10 CV) was performed. Protein samples were eluted with 3 CV of TBS-E (50 mM Tris HCl pH 7.4, 400 mM NaCl, 250 mM imidazole, 10% glycerol). The protein was aliquoted to 1 ml aliquots in Eppendorf tubes, frozen in liquid nitrogen and stored at –80 °C.

Purification of DD-ligand complexes

Size exclusion chromatography was performed for the further purification of the complexes of DD fusion constructs with their targets (MBP, GFP). The DD fusion construct was mixed with MBP or GFP at 1:1 molar ratio in HBS_{150} (10 mM Hepes pH 7.4, 150 mM NaCl) and then applied to a preparative Superdex-200 column (Amersham Pharmacia) on an Äkta Prime system (GE Healthcare).

For every protein mixture, the peak fraction with the smallest molecular weight containing both DD fusion and its target in equimolar amounts, as determined by SDS-PAGE, was collected and used for crystallization. All steps were performed at 4 °C.

ELISA

DD fusion constructs were tested by ELISA to qualitatively assess binding to individual targets. In this setup 100 µl of 1 µM biotinylated target protein (MBP, GFP) was immobilized on a Maxisorp plate pre-coated with Neutravidin and the plate was incubated with 100 µl of 200 nM DD fusion constructs, carrying an N-terminal $RGSHis_6$ tag. Binding was detected with anti-RGS-His-antibody-HRP conjugate (QIAGEN) by measuring the absorbance at OD_{450}.

Crystallization

Eight commercially available grid screens of the in-house Protein Crystallization Center[§] were used for initial crystallization. The protein was concentrated to 10 - 25 mg/ml using an Amicon Ultra Centrifugal Filter Device (Millipore, USA) with a molecular weight cutoff of 10,000 Da. CrystalQuick crystallization plates (Greiner Bio-One) were used for the sitting drop method. The protein was mixed with the mother liquor in a volume ratio of 1:1, 1:2 and 2:1 for each single condition. Crystal growth took place at 20 °C.

Manual crystallization setups were performed with sitting-drop crystallization plates from Hampton Research. 500 µl reservoir solution was applied and the drop was mixed with the mother liquor in a volume ratio of 1:1, 1:2 and 2:1 for each single condition.

[§] www.bioc.uzh.ch/research/core-facilities/proteincrystallization-center/experimental-setup/initial-xtal-screens/

Data collection and processing

Data were collected from single, cryo-cooled crystals at beamlines PX and PXIII (Swiss Light Source, Villigen, Switzerland) with PILATUS 6M, PILATUS 2M high-resolution diffractometers. Data were processed and scaled with XDS[1].

To find the precise position of the domains, molecular replacement was performed with the following models as separate search models: DARPin off7 (extracted from PDB ID 1SVX), MBP (extracted from PDB ID 1SVX), GFP (PDB ID 1GFL) and designed models of DD constructs, omitting the shared helix itself in the search model. The helix was subsequently built into the density visible between the two DARPin units.

Analysis of the structures

3D alignments of experimental structures to designed models and the calculation of rmsds were performed using the "fit" command within PyMOL on defined selections of Cα atoms[¶]. Crystal-packing interfaces and intermolecular interactions including H-bonds and salt bridges were analyzed[2] with the PDBePISA server[§]. The contributions of individual residues to the interfaces were determined as the difference of the residue solvent accessible surfaces in isolated chains and contact pairs using NACCESS[‡] and custom EXCEL macros. Contact lists were generated custom EXCEL macros.

Analysis of crystal contacts

For DARPin *D12* and constructs *D12*-H02-*D12*, *D12*-H06-*D12*-H06-*D12*, *D12*-H09-*D12*, *D12*-H12-*D12*-H12-*D12* ($P2_12_12_1$), *D12*-H12-*D12*-H12-*D12* ($P3_121$), *D12*-H15-*D12* and *D12*-H15-*D12*-H15-*D12* crystal packing was dominated by *D12* paratope-paratope contacts in various relative orientations, while in *D12*_H11_*D12*, *D12*_H13_*D12* and *D12*-H12-*D12*-H12-*D12* ($P2_1$) paratope-capping repeat and paratope-linker module interactions prevailed. To analyze these contacts, all symmetry-related molecules in contact with the asymmetric unit were generated and the crystal contacts scrutinized. To analyze the paratope-paratope interactions, the two DARPin modules involved in the contact were copied into a new object and 3D-aligned by either one or the other of the two DARPins, then clustered according to the different relative orientations of the DARPins (Supplementary Figure 4).

¶ www.pymol.org

§ www.ebi.ac.uk/pdbe/pisa/

‡ http://www.bioinf.manchester.ac.uk/naccess/

Deviations from the design are induced by crystallization artefacts

Three structures out of fourteen showed significant deviations from the design (Supplementary Figure 4)**.** Structure *D12*_H09_*D12* shows the largest deviation (rmsd 7.0 Å), as residues 141-156 (AQDKFGKTPRDLARDN) between the third internal repeat of the first DARPin and the beginning of the shared helix are disordered in all six chains of the asymmetric unit. The shared helix assumes an orientation that is almost at a 90° angle to the designed orientation (Supplementary Fig. 4a), resulting in a rotation of the second DARPin around its long axis compared to the model. Analysis of the crystal packing revealed the cause of this deviation: four copies of the protein arrange in a barrel-shaped bundle. Pairs of chains interact through both *D12* paratopes of the DD construct, resulting in a crystal interface with hydrophobic contacts that would not be possible for the designed conformation. Thus, the intramolecular interactions of the shared helix with the N-terminal DARPin are perturbed in favor of strong intermolecular interactions.

Interestingly, the structure of the second construct with a shared helix H09 also deviates from the initial design, but in a different manner. Construct *off7*_H09_*3G124* (rmsd 6.0 Å) crystallized without target at pH 4.6. DARPins harbor a conserved histidine in each repeat that forms hydrogen bonds to the backbone of two adjacent loops. This hydrogen bond network involves all internal repeats, spanning the entire paratope of the DARPin[3]. Protonation of histidine at low pH would interfere with this network, thus destabilizing the DARPin and interfering with ligand binding. In the unliganded *off7*_H09_*3G124* structure (crystallized at pH 4.6), crystal packing interactions can contribute to the perturbed interactions between the internal repeats of the N-terminal DARPin and the shared helix (Supplementary Fig. 4c).

In the *D12*_H11_*D12* structure (rmsd 6.4 Å) the shared helix also shows a marked kink (Supplementary Fig. 4b). As a result, the structure of the C-terminal DARPin is shifted by one repeat towards the C-terminal end. Again, crystal packing offers an explanation. Lys144 in the connector loop of a symmetry mate interacts with Glu166 and Lys167 on one side of the helix kink, the neighboring Phe145 binds between Leu170 and Leu171 on the other side of the kink, and Val111 and Ile112 of the symmetry mate reinforce the hydrophobic interaction. The *off7*:MBP_H11_*3G124*:GFP complex structure does not form this crystal contact and agrees fairly well with the design (rmsd 1.7 Å).

Supplementary References

1. Kabsch W. Xds. *Acta Crystallogr. D Biol. Crystallogr.* **66**, 125-132 (2010).
2. Krissinel E. & Henrick K. Inference of macromolecular assemblies from crystalline state. *J. Mol. Biol.* **372**, 774-797 (2007).
3. Kohl A., Binz H. K., Forrer P., Stumpp M. T., Plückthun A. & Grütter M. G. Designed to be stable: crystal structure of a consensus ankyrin repeat protein. *Proc. Natl. Acad. Sci. U. S. A.* **100**, 1700-1705 (2003).
